# Assessment of Polychlorinated Biphenyls and Their Hydroxylated Metabolites in Postmortem Human Brain Samples: Age and Brain Region Differences

**DOI:** 10.1021/acs.est.2c00581

**Published:** 2022-06-03

**Authors:** Xueshu Li, Marco M. Hefti, Rachel F. Marek, Keri C. Hornbuckle, Kai Wang, Hans-Joachim Lehmler

**Affiliations:** † Department of Occupational and Environmental Health, University of Iowa, Iowa City, Iowa 52242, United States; ‡ Department of Pathology, University of Iowa Hospital and Clinics, Iowa City, Iowa 52242, United States; § IIHR-Hydroscience and Engineering, University of Iowa, Iowa City, Iowa 52242, United States; ∥ Department of Biostatistics, University of Iowa, Iowa City, Iowa 52242, United States; ⊥ Department of Civil and Environmental Engineering, University of Iowa, Iowa City, Iowa 52242, United States

**Keywords:** brain region, gas chromatography−tandem mass spectrometry, hydroxylated polychlorinated biphenyls, liquid chromatography−high resolution mass spectrometry, neurotoxicant, polychlorinated biphenyls

## Abstract

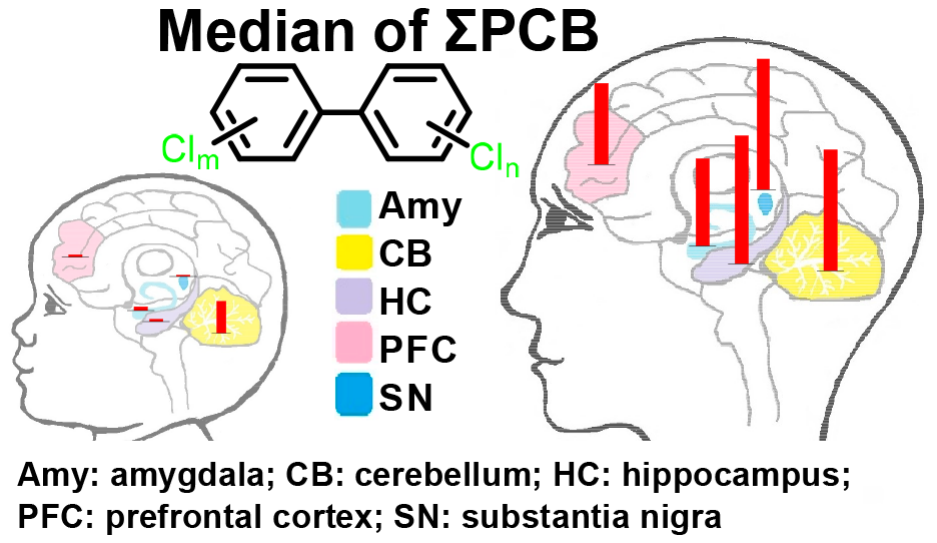

Exposure to polychlorinated biphenyls (PCBs) and their hydroxylated metabolites (OH-PCBs) has been implicated in neurodevelopmental disorders. However, the distribution of PCBs and OH-PCBs in the human brain has not been characterized. This study investigated the age-, sex-, and brain region-specific distribution of all 209 PCBs using gaschromatography–tandem mass spectrometry (GC–MS/MS) in neonatal (*N* = 7) and adult (*N* = 7) postmortem brain samples. OH-PCB analyses were performed by GC–MS/MS (as methylated derivatives) and, in a subset of samples, by nontarget liquid chromatography high-resolution mass spectrometry (Nt-LCMS). Fourteen higher chlorinated PCB congeners were observed with a detection frequency >50%. Six lower chlorinated PCBs were detected with a detection frequency >10%. Higher chlorinated PCBs were observed with higher levels in samples from adult versus younger donors. PCB congener profiles from adult donors showed more similarities across brain regions and donors than younger donors. We also assess the potential neurotoxicity of the PCB residues in the human brain with neurotoxic equivalency (NEQ) approaches. The median ΣNEQs, calculated for the PCB homologues, were 40-fold higher in older versus younger donors. Importantly, lower chlorinated PCBs made considerable contributions to the neurotoxic potential of PCB residues in some donors. OH-PCBs were identified for the first time in a small number of human brain samples by GC–MS/MS and Nt-LCMS analyses, and all contained four or fewer chlorine.

## Abstract 2

PCB detection frequencies and levels are age- and brain region-dependent, whereas hydroxylated PCBs have low detection frequencies in postmortem human brain tissues.

## Introduction

Polychlorinated biphenyls (PCBs) were used in building materials and fluorescent light ballasts until their production was banned in the United States in the late 1970s.^1,2^ They are still produced inadvertently and are present in consumer products, such as paints and polymer resins.^3−5^ The release of PCBs from these diverse uses has resulted in their ubiquitous presence in the environment. PCB congener profiles change as PCBs move through aquatic and terrestrial food chains,^6,7^ resulting in human exposures to mostly higher chlorinated PCB congeners via the diet.^8^ The PCB residue levels in the environment and humans have decreased over the last four decades.^6,7,9,10^ Some of the inadvertently produced PCB congeners, such as PCB 11, are not present in the original technical PCB mixtures^11^ but are detected in the environment, foodstuff, and humans.^12−15^ Moreover, inhalation exposure, especially to lower chlorinated PCBs, is increasingly recognized as a route of human exposure.^16−18^

Only limited information about PCB congener profiles and levels in human tissue samples is available, especially on a congener-specific level. These studies are several decades old, use analytical methods that lack sensitivity and selectivity, and quantify only a few selected marker PCB congeners. Despite these limitations, the available literature provides general insights into the distribution of PCBs in human tissues. Like in animal studies,^19−21^ PCB tissue levels in humans directly correspond to tissue lipid levels because of the lipophilicity of PCBs. For example, PCB concentrations in serum are much lower than those in human adipose tissue.^22−24^ The brain is an exception to this rule due to the blood–brain barrier and the unique lipid composition of the brain. For example, levels of PCB congeners are higher in the subcutaneous fat than in the brain in the autopsy samples from Greenland^25^ and Finland.^26^ The Finnish study reported that the total PCB levels were higher in adipose tissue compared to the liver and brain.^26^

Exposure to PCBs has been linked to cancer, cardiovascular disease, and other adverse outcomes.^2^ PCBs cause adverse neurodevelopment outcomes and, possibly, neurodegenerative diseases.^27−29^ Neurotoxic outcomes were first reported for patients from the Yusho PCB poisoning event.^30^ Since these early reports, laboratory and epidemiological studies have demonstrated associations of PCB exposures with adverse neurodevelopmental outcomes.^31,32^ PCBs are also implicated in neurodegenerative diseases, such as Parkinson’s disease.^28^ PCBs are potent neurotoxicants across the lifetime; however, limited information is available about PCB profiles and levels in human brain tissues, especially lower chlorinated PCBs that are emerging as a human health concern, and their distribution in different brain regions.

PCBs are oxidized by cytochrome P450 enzymes to hydroxylated PCBs (OH-PCBs) in humans.^33,34^ Several studies suggest that PCB congeners are preferentially oxidized to meta- and para-hydroxylated metabolites by human cytochrome P450 enzymes.^35−38^ Some OH-PCB congeners are selectively retained in human blood^39^ and are present in human populations .^40−43^ Other, mostly lower chlorinated, PCBs are readily eliminated, either as free OH-PCBs or OH-PCB conjugates.^44−46^ Levels of OH-PCBs in human blood and placenta have received considerable attention, and epidemiological studies implicate OH-PCBs in adverse health outcomes, including neurotoxic outcomes.^33^ For example, maternal exposure to OH-PCBs during pregnancy may increase neonatal T4 levels.^47^ Laboratory studies also implicate OH-PCBs in neurotoxic outcomes.^33,48^ While OH-PCBs have been reported in animal brain tissue,^49,50^ it is currently unknown whether OH-PCBs are present in the human brain.

This study characterized the PCB and OH-PCB profiles and levels in postmortem human brain samples. We hypothesized that there are age- and brain region-specific differences in PCB profiles and levels, with PCB signatures in the neonatal brain being distinctively different from those observed in the adult brain. Moreover, we assessed for the first time whether or not OH-PCBs are present in the human brain to provide critically needed insights for preclinical and epidemiological studies of neurodevelopmental or neurodegenerative disorders linked to PCB exposure.

## Experimental Section

### Reagents and Materials

Postmortem brain tissues, including the amygdala (*N* = 13), Brodmann area 19 (BA19; *N* = 11), cerebellum (*N* = 13), hippocampus (*N* = 10), prefrontal cortex (*N* = 8), and substantia nigra (*N* = 9), were obtained from the Iowa Brain Bank, Iowa City, Iowa. These brain regions were selected because of their potential role in PCB developmental neurotoxicity or because they have been studied previously.^51^ All samples were collected in 2017 and 2018 from seven donors aged 0 days to 1 year (6 female and 1 male) and seven donors aged 58 to 80 (4 female and 3 male). Tissue samples were flash-frozen at the time of brain removal and stored at −80 °C. A summary of donor information is provided in Table S1. The University of Iowa Hawk IRB reviewed the protocol for collecting brain samples by the Iowa Brain Bank. It was determined not to constitute human subjects research because it involves tissue exclusively from deceased subjects (Determination #201706772). Retention and use of tissue for research from these deceased donors are authorized by the next of kin as part of the autopsy consent process and comply with all applicable state and federal laws and regulations.

### PCB and OH-PCB Nomenclature and Sources

The PCB names followed the EPA nomenclature for PCBs.^52^ The OH-PCB congener nomenlature (Table S2) uses previously published abbreviations for PCB metabolites.^53^ Briefly, this nomenclature reports only the position of the OH group and the PCB congener number. For the sources of analytical standards and other materials, see the Supporting Information.

### Extraction of PCBs and OH-PCBs from Brain Tissues

PCBs and OH-PCBs were extracted from brain tissues using published extraction methods with modifications.^18,20^ Briefly, brain tissues (0.3–1.2 g) and 3 mL of isopropanol were placed in medium glass tubes, followed by homogenization of the tissue for 30 s with a TissueRuptor (Qiagen, Hilden, Germany). Then, PCBs and OH-PCBs were extracted from the tissue homogenates using a published method^20,41^ with modification, as described in the Supporting Information.

### Gas Chromatographic Determinations

PCB samples were analyzed on an Agilent 7890A GC system coupled with an Agilent 7000 Triple Quad and an Agilent 7693 sampler (GC–MS/MS) in the multiple reaction monitoring mode (MRM) on an SPB-Octyl column (30 m length, 250 μm inner diameter, and 0.25 μm film thickness; Sigma-Aldrich, St. Louis, MO, USA). This analytical method allows the quantification of 209 PCB congeners as 174 individual or co-eluting chromatographic peaks. OH-PCB samples, as methylated derivatives, were analyzed on an Agilent 7890B system coupled with an Agilent 7000D Triple Quad and an Agilent 7693 sampler in the MRM mode using an SPB-Octyl column. Confirmatory OH-PCB analyses were performed with a subset of extracts using a DB-1701 column (30 m length, 250 μm inner diameter, and 0.25 μm film thickness; Sigma-Aldrich). This analytical method allows the quantification of 72 MeO-PCBs as 66 and 67 peaks of individual or co-eluting peaks on the SPB-Octyl and DB-1701 columns, respectively. The temperature program and instrument parameters for the PCB and OH-PCB analyses are summarized in the Supporting Information. PCB and OH-PCB levels were corrected for recovery levels of an appropriate surrogate standard on a per-sample basis (Table S3) and adjusted for tissue wet weight because the gravimetrically determined extractable lipid content poorly predicts PCB partitioning into the brain.^57^ Moreover, the brain lipid composition changes with age^54^ which may affect PCB partitioning into the brain. We also calculated neurotoxic equivalent quotients (NEQs) by multiplying the tissue concentration with the published neurotoxic equivalency factors (Table S10).^55^

### Quality Assurance/Quality Control

The extraction efficiency, reproducibility, and accuracy of the GC–MS/MS analyses were assessed using ^13^C-labeled surrogate standards (Table S3), method blanks, and a standard reference material (SRM 1957, NIST), as described in the Supporting Information. Limits of detection (LODs) were calculated from the method blanks and are summarized in Tables S4 and S5.^56^ For PCB and OH-PCB levels in SRM 1957, see Tables S6 and S7.

### Statistical Analysis of GC–MS/MS Data

All data, including the results from the GC–MS/MS analyses and the NEQ values, are presented as mean ± standard deviation (Tables S8–S12). The statistical analyses of grouped PCBs and NEQs were assessed with one-way ANOVA (Tables S13 and S14). Differences in the detection frequencies and levels of PCB congeners were analyzed with Fisher’s exact test or the Tobit regression model, respectively (Table S15). These statistical analyses were performed with R (version 3.6.3). The PCB profiles were compared with the similarity coefficients cos θ, as described (Tables S16 and S17).^57^

### PCB Metabolite Extraction for Nt-LCMS Analysis

A subset of brain tissue samples *N* = 18) was selected for Nt-LCMS screening for OH-PCB using a modified protocol based on earlier studies.^58−60^ Briefly, human brain tissues (∼0.5 g) were homogenized in glass tubes with 2 mL of Milli-Q water using a TissueRuptor (Qiagen). The homogenates were spiked with 4′-chloro-3′-fluoro-4-hydroxy-biphenyl and the corresponding PCB sulfate (100 ng of each) in acetonitrile. Acetonitrile with 1% formic acid (2 mL) was added to the tubes, and samples were vortexed for 10 s, followed by the addition of 200 mg of sodium chloride and 800 mg of magnesium sulfate. The samples were shaken vigorously, inverted for 5 min, and centrifuged at 1181 g for 5 min to facilitate the phase separation. The top organic phases were passed through hybrid phospholipid solid-phase extraction (HybridSPE) cartridges (3 mL, Millipore Sigma, Burlington, Massachusetts, USA) loaded with 3 g of a mixture of anhydrous sodium sulfate and anhydrous magnesium sulfate (1:1, w/w). The aqueous phases were re-extracted with 1 mL of acetonitrile, and the organic phase was passed through the HybridSPE cartridge. The HybridSPE cartridges were washed with an additional 3 mL of acetonitrile. The combined eluents were evaporated to dryness with a Savant SpeedVac SPD vacuum concentrator with an RVT5105 refrigerated vapor trap (Thermo Scientific, Waltham, Massachusetts, USA) at 35 °C; the residues were redissolved in 200 μL of acetonitrile and transferred to centrifuge tubes. The samples were again evaporated to dryness with a SpeedVac concentrator. The extract was reconstituted in 200 μL of acetonitrile–water (1:1, v/v), and 100 ng of perfluorooctanesulfonic acid in acetonitrile was added as the internal standard. The extracts were centrifuged at 16 000 g and 4 °C for 10 min, and the supernatants were transferred to autosampler vials and kept at −80 °C.

### Nt-LCMS Determination of OH-PCBs

Brain extracts were analyzed on a Q-exactive Orbitrap mass spectrometer (Thermo Fisher Scientific, Waltham, MA, USA) with a Vanquish Flex ultra-high-performance liquid chromatograph (Thermo Fisher Scientific) with an ACQUITY UPLC-C18 column (particle size: 1.7 μm, 2.1 × 100 mm, Waters, Milford, MA, USA) at the High-Resolution Mass Spectrometry Facility of the University of Iowa. A description of chromatographic separation, instrument parameters, and the data processing procedure is provided in the Supporting Information. Solvent (50% water/50% acetonitrile) and method blanks were used to monitor the carryover. No OH-PCB metabolites were detected in these blank samples. The average recoveries of 4′-chloro-3′-fluoro-4-hydroxy-biphenyl and the corresponding F-tagged PCB sulfate were 91 ± 10% (range, 74–106%) and 77 ± 14% (range, 58–110%), respectively. Detailed Nt-LCMS results are summarized in Table S18.

## Results and Discussion

### Detection Frequencies of PCBs in the Human Brain

**Figure 1:**
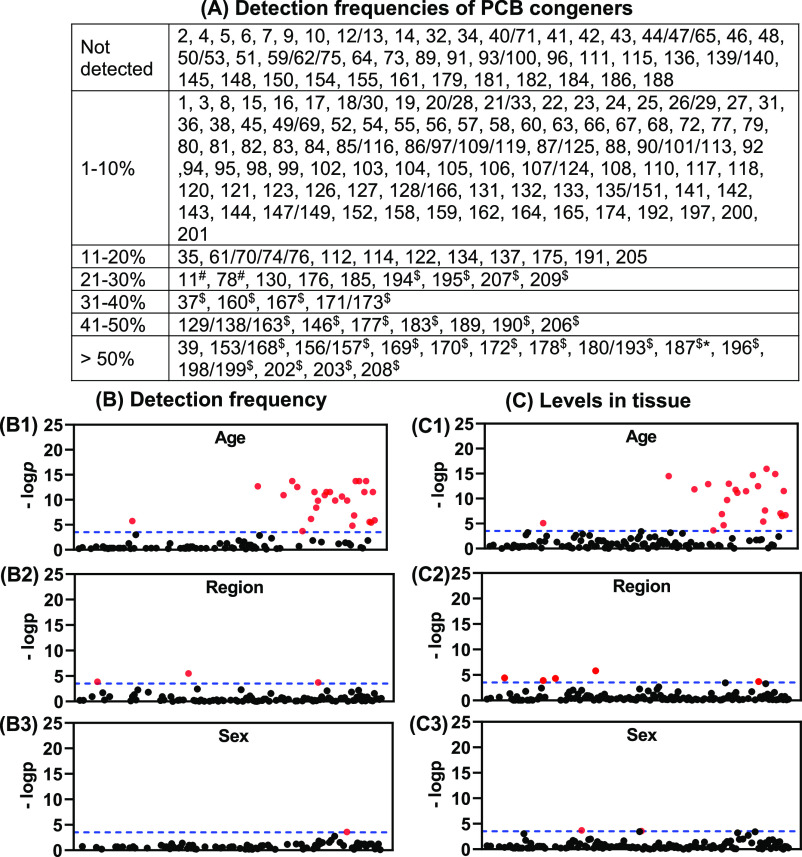
(A) Detection frequencies of PCB congeners reveal age- and brain region-dependent differences. Plots of *p*-values comparing detection frequencies (B) and levels (C) of single or co-eluting PCB congeners between two age groups (B1 and C1), seven brain regions (B2 and C2), and two sex groups (B3 and C3) also show significant differences based on age, brain region, and sex. The dotted line indicates the—log *p* values of Bonferroni-adjusted multiple comparisons (*p* = 2.89 × 10^–4^). Detection frequencies were determined in 63 tissue samples from seven brain regions for all 209 PCB congeners, analyzed as 173 peaks of single or co-eluting congeners. Red dots in panels (B,C) indicate PCB congeners with a significant difference. PCB congeners are plotted on the x-axis in the order of their Ballschmiter and Zell number, as defined by the EPA.^52^ Congeners with *p* = 1 were not plotted. The *p* values of the corresponding PCB congeners are presented in Table S15 and are significant different (*p* < 2.89 × 10^–4^) between age groups (^$^), across brain regions (^#^), and between sex groups (*).

We assessed the detection frequency of all PCB congeners in postmortem brain samples from 14 donors from Iowa by age, sex, or brain region (Figure 1). One lower and 17 higher chlorinated individual or co-eluting PCB congeners were detected in >50% of all brain samples investigated. In addition, 3 lower and 20 higher chlorinated individual or co-eluting PCB congeners were detected in 21-50% of the brain samples. Congeners frequently detected included PCB 202, the most potent ryanodine receptor (RyR)-active PCB congener,^61^ and PCB 169, an aryl hydrocarbon receptor (AhR) agonist.^62^ PCB 153 and PCB 180 are two frequently detected PCB congeners that have also been detected in human brain tissue in earlier studies.^25,51,63,64^

The observation that higher chlorinated PCBs are predominant in human brain tissue is not entirely surprising. These congeners are important constituents of higher chlorinated technical PCB mixtures.^65^ Moreover, PCB patterns shift from lower to higher chlorinated PCBs in food chains because lower chlorinated PCBs, especially congeners without para-chlorine groups, are typically more rapidly metabolized by cytochrome P450 enzymes than higher chlorinated PCBs, in particular those with a 2,4,5-trichloro-substitution pattern. For example, PCB 11, but not penta- and hexachlorinated PCBs, is metabolized in HepG2 cells in culture.^37,66^ Historically, humans were primarily exposed to higher chlorinated PCB via the diet;^67^ however, current exposures to PCBs occur via the diet and by inhalation.^8,17,18^

Information about the prevalence of PCB congeners in human brain samples is limited to a few studies and typically focuses on persistent congeners. One of the more robust PCB studies in human brain tissue measured 14 PCB congeners in postmortem brain (*N* = 17) and other tissues collected in 1994 in Greenland.^25^ Detection frequencies of the 14 PCB congeners ranged from 6 to 100%, with PCB 99, PCB 118, PCB 138, PCB 153, PCB 156, PCB 170, PCB 180, and PCB 187 being the most abundant PCB congeners in the brain. These congeners with a 2,4,5-trichloro-substitution pattern are predominant PCB congeners in humans.^68^ In contrast, the detection frequencies of two lower chlorinated PCBs, PCB 28 and PCB 52, were found in 41 and 28% of brain samples from Greenland, respectively.^25^ Both the PCB congeners are important constituents of technical PCB mixtures^65^ and, based on a recent study, are prevalent in some at-risk populations.^69^ The lower detection frequency of PCB 52 in the earlier study is consistent with its metabolism by human cytochrome P450 enzymes.^70^ In our study, the detection frequencies of PCB 129/138/163, PCB 153/168, PCB 156/157, PCB 170, PCB 180/193, and PCB 187 ranged from 49 to 56% (Table S9). These congeners were typically present in the brain of older but not younger donors (91–94% versus 0–10%, respectively; −log *p* > 3.539, Table S15). Both PCB 20/28 and PCB 52 were only detected in one cerebellum sample from a female, 0-day-old donor (2% detection frequency).

There has been some interest in the levels of PCB 95, a RyR-active, neurotoxic PCB congener,^71−74^ in the brain of rodents and humans. In our study, PCB 95 had a detection frequency of 3% and was only detected in two brain regions from one donor, an 80-year-old female. In an earlier study, PCB 95 was detected by gas chromatography with electron capture detection in 11% of brain samples, and the detection frequency was significantly associated with neurodevelopmental disorders.^51^ A reanalysis of a subset of extracts from this earlier study by GC–MS confirmed the presence of PCB 95 in the earlier study.^75^ PCB 95 was abundant in maternal serum from a cohort of pregnant women at an increased risk for having a child with a neurodevelopmental disorder.^69^ Two lower chlorinated PCB congeners, PCB 11 and PCB 28, were the major PCB congeners detected in this cohort of pregnant women.^15,69^ Our findings suggest that, in contrast to the two earlier studies, current environmental exposures to PCB 95 are low, or PCB 95 is rapidly eliminated; however, additional studies are needed to confirm either hypothesis.

There is a significant knowledge gap regarding lower chlorinated PCB congeners in human tissue. In our study, we found six lower chlorinated PCB congeners (≤4 chlorines), including PCB 11, PCB 35, PCB 37, PCB 39, PCB 61, and PCB 78, with detection frequencies > 10% in the brain samples. Of particular interest is the presence of PCB 11, a PCB congener that, for example, is formed inadvertently during the production of paint pigments.^4^ This PCB congener is present in the environment^13,76^ and foodstuff^14^ and shows neurotoxic potential based on the studies in cells in culture.^15,77^ Importantly, human biomonitoring studies demonstrate that the US population, including Iowans, are exposed to PCB 11.^69,78^

### Age, Sex, and Brain Region Dependence of the PCB Detection Frequencies

We observed significant differences in the detection frequencies of PCBs by age and, to a lesser extent, by sex and brain region (Figure 1 and Table S15). Overall, 26 higher chlorinated PCB congeners were more frequently detected in tissues from older than younger donors. The detection frequencies of PCB 11, PCB 78, and PCB 169 displayed statistically significant differences across brain regions. PCB 11 was detected most frequently in the cerebellum and Brodmann area 19 samples, and PCB 78 was most frequently detected in the cerebellum. In contrast, PCB 169 was present in most brain regions (≥50% of the brain region samples analyzed) but had a low detection frequency in the prefrontal cortex. An earlier study found no significant differences in PCB levels in cortex versus cerebellum from postmortem donors; however, this observation needs to be interpreted with caution because only eight PCB congeners were analyzed using a nonselective electron capture detector.^51^ A study in weanling rats demonstrated the nonuniform distribution of individual PCB congeners in the brain, suggesting that differences in the composition of brain regions influence the partitioning of PCBs in different brain regions.^79^

### Levels of PCBs in Different Human Brain Regions

**Figure 2:**
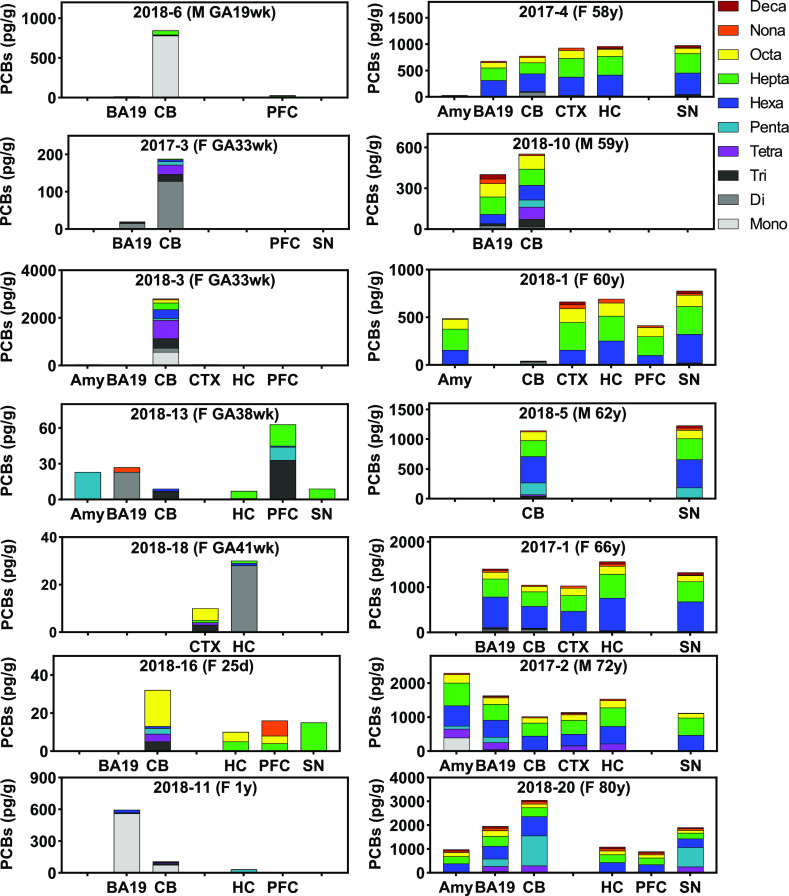
PCB levels and PCB homologue compositions in different brain regions show clear differences depending on age and brain region. Overall, PCB levels in neonatal samples (<1-year-old; left column) were lower compared to the samples from adults (58–80 years old; right column). Moreover, PCB residues in the brain of adult donors contained predominantly hexa- to octachlorinated PCBs. In contrast, PCB residues in neonatal brains showed a more complex PCB homologue profile than the adult samples. Also, the PCB homologue in brain regions from the same donor showed more variability in neonatal than adult brain samples. PCB levels were adjusted by wet brain tissue weight (pg/g ww) in different human brain regions from two age group donors. GA, gestational age; Amy, amygdala; BA19, Brodmann area 19; CB, cerebellum; CTX, cortex; HC, hippocampus; PFC, prefrontal cortex; SN, substantia nigra.

PCB levels in brain regions were analyzed by the sum of PCBs (ΣPCBs), PCB homologue group, class, number of ortho-chlorine substituents, and congeners to assess differences by age, sex, or brain region (Figure 2 and Table S8). Homologue groups were analyzed because the metabolism and, consequently, elimination of PCBs decrease with the degree of chlorination (i.e., the homologue group).^80^ We also assessed levels by PCB class, which takes structural features involved in PCB metabolism into account that relate to their persistence in vivo and their toxicity.^81,82^ In this paper, PCB classes are defined as (A) congeners with 4-, 3,4-, and 3,4,5-substitution patterns and zero or one ortho-chlorine substituent; (B) congeners with two or more ortho-chlorine substituents and a 2,4- or 2,3,4-substitution pattern; (C) PCB congeners with a 2,4,5-substitution pattern; and (D) episodic PCB congeners^83^ that are rapidly eliminated following exposure. The number of ortho-chlorine substituents, and also the homologue groups and PCB class, identifies structural features that are linked to the toxicity of PCBs.^84^

The ΣPCB levels in the different brain regions were variable and ranged from 32 to 3050 pg/g ww (average: 1084 ± 615 pg/g ww) in the older donors and from not detected (ND) to 2802 pg/g ww (average: 191 ± 567 pg/g ww) in the younger donors (Table S8). PCB 153/168, PCB 180/193, and PCB 202 were the major PCB congeners. The maximum level of PCB 202, the most potent RyR-active PCB congener,^61^ was 34 pg/g ww. For comparison, the serum levels of PCB 202 in another Midwestern population ranged from < LOQ to 12.5 pg/g ww.^43^ PCB 138, PCB 153, PCB 170, and PCB 180 were the major congeners (out of 24 congeners analyzed) detected in a single brain sample from Belgium.^63^ In the Belgian brain sample, PCB 95 was not detected, and levels of individual PCBs ranged from ND for several congeners to 3100 pg/g ww for PCB 153. Several earlier studies also reported that PCB 138, PCB 153, and PCB 180 were the major PCB congeners in the human brain. However, comparing these studies is challenging because the PCB levels were lipid-adjusted, and only a few PCB congeners were analyzed.^25,51,64^

PCB levels in the neonatal brain have received little attention to date. In the present study, levels of hexa- to decachlorinated PCBs, class B and class C PCB congeners, and many PCB congeners with ≥ 2 chlorine substituents were significantly lower in brain tissue samples from younger compared to older donors (Figure 1 and Table S8). PCB congeners falling into these categories are typically more persistent in humans and accumulate with age. In addition, neonates have a different lipid composition in the first weeks after birth,^54^ which may also contribute to the differences in PCB levels between younger versus older donors. Levels of class A PCBs differed significantly by the brain region (*p* = 0.0323), with mean levels of 194 ± 293 pg/g tissue in the cerebellum (*N* = 13). In contrast, the mean levels of class A PCBs in other brain regions ranged from 25 ± 22 in the prefrontal cortex (*N* = 8) to 101 ± 183 pg/g tissue in the amygdala (*N* = 6). Levels of class B (292 ± 136 pg/g tissue in old versus 35 ± 81 pg/g tissue in young donors; *p* < 0.0001) and class C congeners (595 ± 224 pg/g tissue in old versus 56 ± 153 pg/g tissue in young donors; *p* = 0.0001) differed significantly by age (Table S8).

Consistent with our analysis of the detection frequencies, the levels of most persistent congeners showed significant differences by age (Figure 1 and Table S9). For example, in the older donors, mean levels were 157 pg/g ww (ND to 295 pg/g ww) for PCB 129/138/163 and 183 pg/g ww (ND to 310 pg/g ww) for PCB 153/168. The detection frequencies and levels of both PCB congeners were lower in the brain tissues of younger donors. PCB129/138/163 and PCB 153/168 were detected in one out of 30 brain samples from the younger donors, with levels of 140 and 164 pg/g ww, respectively. While our study is the first to report PCBs in the brains of human neonates, age-dependent changes in PCB levels in human tissues have been documented in several other studies. Levels of persistent PCBs in postmortem human brain samples from Greenland increase with age.^25^ Levels of three higher chlorinated PCB congeners, PCB 118, PCB 138, and PCB 153, in adipose tissue from Korea also increased with age.^85^ Similarly, serum PCB levels increase with age in adult populations.^44.^ No significant association with age was observed for the levels of PCB 28 and PCB 52 in the Korean study. Significant differences in the PCB 20/28 and PCB 52 levels by age were not observed in this (Table S9) and another population from Iowa, United States; however, different factors contribute to the differences in PCB levels with age.^43^ In adult populations from across the world, PCB levels increase with age due to the bioaccumulation of persistent PCBs following dietary and other exposures. In contrast, neonates do not have a lifetime of PCB exposure and have an immature hepatic cytochrome P450 system.^86,87^ Thus, neonates may accumulate PCB congeners that would be rapidly eliminated in adults.

Although levels of the sum of class A congeners differed by sex, only individual class A congeners PCB 63 (−log *p* = 3.71) and PCB 107/124 (−log *p* = 3.58) showed a significant difference by sex in this study (Table S15 and Figure 1c3). Several other studies report conflicting sex differences in human tissue samples. PCB levels in postmortem human tissue samples from Greenland showed no statistically significant differences by sex.^25^ In paired tissue and serum samples from Finland, the partitioning of PCBs was independent of sex.^22^ However, higher PCB serum levels were found in the Finnish study in female than male adults (*n* = 116; p = 0.03). In contrast, higher total PCB levels were reported in males than females in adipose tissue for the general US population.^88^ Significant sex differences were also observed for PCB 118 in the adipose tissue (males > females) and PCB 138 in the liver in autopsy samples from Korea.^85^ The inconsistent sex differences in PCB levels are not surprising because sexual dimorphisms are not consistently observed in humans.^89^ Thus, PCB tissue levels in humans may result from complex human exposure. Similarly, many rodent studies report no sex differences in the tissue levels of PCBs,^49,90−92^ despite reports that the metabolism of some PCB congeners is sex-dependent.^93^

### NEQs in the Human Brain

We used the updated NEQ approach from the study of Holland et al. (2021) to assess the potential neurotoxicity of the PCB residues detected in the human brain (Table 1; for other NEQ estimations, see Table S10).^55^ The median ΣNEQs based on the multiple mechanism NEQ scheme for PCB homologues were 40-fold higher in older versus younger donors, with ΣNEQs of 290 pg/g ww (ranged from 5 to 858 pg/g ww) and 7 pg/g ww (ranged from ND to 732 pg/g ww), respectively. In brain samples from older donors, hexa- to octachlorinated PCBs had relatively high median NEQs. Interestingly, monochlorinated PCBs also had a relatively high median NEQ in the older donors (median NEQ of 12 pg/g ww, ranging from ND to 22 pg/g ww). Monochlorinated PCBs had the highest median NEQ in younger donors (median NEQ of 31, ranging from ND to 44). Across all donors, ΣNEQ varied considerably between brain regions. Comparable median ΣNEQs were observed in brain regions that may play a role in PCB developmental neurotoxicity, including the cerebellum, cortex, hippocampus, and substantia nigra, ranging from 182 in the cerebellum to 295 in the substantia nigra. The lowest median ΣNEQ of 12 was observed in the prefrontal cortex. Higher chlorinated PCBs had high median NEQs across all brain regions. However, monochlorinated PCBs had comparatively high median NEQs in the amygdala, BA19, and the cerebellum. These observations are consistent with the significant age and brain region differences observed based on the detection frequency and the PCB levels (Figure 2). Of particular importance is the finding that lower chlorinated PCBs can considerably contribute to the neurotoxic potential of PCB residues in the human brain, as assessed using the NEQ approach. While there is some in vitro evidence that lower chlorinated PCBs are developmental neurotoxicants,^69,94^ the neurotoxicity of lower chlorinated PCBs remains largely unexplored.

**Table 1:** Median and Range of PCB Neurotoxic Equivalents for PCB Homologue Groups and Total PCBs in Postmortem Human Brain Samples, Calculated Based on NEFs for Multiple Mechanisms of Action^a^

PCB homologue	age		gender		brain region						
	older donors (*n* = 33)	younger donors (*n* = 30)	male donors (*n* = 13)	female donors (*n* = 50)	amy (*n* = 6)	BA19 (*n* = 11)	CB (*n* = 13)	CTX (*n* = 6)	HC (*n* = 10)	PFC (*n* = 8)	SN (*n* = 9)
mono	25 (ND, 46)	66 (ND, 91)	69 (ND, 91)	37 (ND, 66)	46 (ND, 0)	35 (ND, 66)	66 (ND, 91)	ND	ND	ND	ND
di	10 (ND, 25)	8 (ND, 50)	8 (ND, 10)	13 (ND, 50)	ND	9 (ND, 10)	17 (ND, 50)	ND	8 (ND, 8)	ND	ND
tri	2 (ND, 13)	1 (ND, 93)	3 (ND, 13)	2 (ND, 93)	1 (ND, 27)	2 (ND, 5)	3 (ND, 93)	1 (ND, 1)	3 (ND, 7)	6 (ND, 8)	4 (ND, 10)
tetra	7 (ND, 94)	3 (ND, 252)	39 (ND, 83)	3 (ND, 252)	83 (ND, 141)	69 (ND, 70)	3 (ND, 252)	2 (ND, 49)	35 (ND, 67)	ND	1 (ND, 80)
penta	4 (ND, 377)	3 (ND, 24)	21 (ND, 60)	3 (ND, 377)	7 (ND, 174)	48 (ND, 94)	1 (ND, 377)	2 (ND, 8)	3 (ND, 9)	2 (ND, 3)	3 (ND, 244)
hexa	93 (ND, 189)	0.2 (ND, 86)	100 (ND, 141)	37 (ND, 189)	82 (ND, 157)	7 (ND, 158)	25 (ND, 189)	81 (ND, 106)	74 (ND, 169)	37 (ND, 76)	90 (ND, 153)
hepta	90 (ND, 174)	1 (ND, 74)	92 (ND, 174)	57 (ND, 137)	58 (ND, 79)	48 (ND, 122)	55 (ND, 100)	84 (ND, 106)	68 (ND, 142)	5 (ND, 74)	85 (ND, 133)
octa	89 (ND, 157)	3 (ND, 87)	93 (ND, 157)	79 (ND, 146)	104 (ND, 104)	92 (ND, 146)	73 (ND, 96)	94 (ND, 102)	93 (ND, 134)	7 (ND, 83)	78 (ND, 89)
ΣNEQ	290 (5, 858)	7 (ND, 732)	325 (ND, 628)	64 (ND, 858)	83 (ND, 274)	74 (ND, 561)	182 (ND, 858)	242 (ND, 340)	214 (ND, 467)	12 (ND, 237)	295 (ND, 545)

a NEQ values for individual tissue extracts were calculated based on the NEFs established for the PCB homologue groups based on multiple mechanisms of action.^55^ Data represent the median of the NEQ values of each homologue group across samples and the ΣNEQ from each sample. For analogous data based on NEFs established for the PCB homologue groups based on a single mechanism of action or NEFs established based on the number of ortho-chlorine substituents and a single or multiple mechanisms of action, see Table S10.

### Comparison of PCB Congener Profiles

The interindividual differences of the PCB congener profiles were compared across donors using the similarity coefficient cos θ, where a value of 0 indicates no similarity and a value of 1 indicates that profiles are identical (Table S16). Across all donors and brain regions, cos θ varied from 0 to 0.99. Typically, older donors had similar PCB profiles (cos θ > 0.5). In contrast, most younger donors had PCB profiles that differ drastically between individuals (cos θ ranged from 0 to 0.93). cos θ also varied from 0 to 0.99 when comparing the PCB congener profiles in different brain regions from the same donor (Table S17). In general, PCB profiles were similar in different brain regions of the older donors (median cos θ = 0.94, ranged from 0 to 1). In contrast, PCB profiles in the brain regions of neonates were more dissimilar (median cos θ = 0.0003, ranged from 0 to 0.67). These findings are consistent with the accumulation of poorly metabolized PCBs in the older donors across their lifespan. In contrast, neonatal PCB exposures appear to be highly variable and quite dissimilar from the PCB residues found in older individuals.

PCB congener profiles in human tissues are rarely reported in the literature, making it challenging to compare our results with other studies. Modest interindividual differences were observed in a small cohort from Sweden (*N* = 7; samples collected before 1998), with the similarity coefficients between individuals ranging from 0.87 to 0.99 in adipose and 0.72 to 0.98 in liver tissues.^95^ The PCB profiles in paired adipose and liver tissue samples were nearly identical in five individuals (cos θ = 0.97 to 1.0) but differed in two male subjects (cos *q* = 0.84 and 0.91). Comparable differences in the similarity coefficients of the PCB congener profiles have been observed in rodent studies following inhalation and oral PCB exposure.^57,96−98^ In the animal studies, these differences in the PCB profiles reflect lower levels of PCB congeners that are more rapidly metabolized.

The comparison of brain PCB profiles provides important lessons for the design of toxicological studies. Neurotoxicity studies of legacy PCBs, either alone or as a mixture, can provide meaningful insights into neurodegenerative outcomes associated with a lifetime of PCB exposure. In contrast, studies of the toxicity of legacy PCBs do not reflect current exposures of neonates and, thus, will not provide insights into the developmental neurotoxicity of PCBs relevant to humans. Moreover, our findings underscore the need to characterize current sources and routes of exposure of neonates to PCBs.

### OH-PCBs in Different Human Brain Regions

We analyzed 72 OH-PCB congeners (as methylated derivatives) in the postmortem brain samples using GC–MS/MS. The OH-PCBs analyzed in this study were selected based on evidence that OH-PCBs formed in humans typically have the hydroxy group in the meta- or para-position.^39^ We analyzed all brain tissue samples using the SPB-Octyl column with a method optimized to analyze OH-PCBs in human serum.^40−43^ In addition, we performed a confirmatory analysis of selected NIST and brain extracts on a DB-1701 column. Although NIST does not publish certified OH-PCB levels, 4-OH-PCB107 and 4-OH-PCB187, the two OH-PCBs commonly detected in human serum,^40,41^ were seen at comparable levels on both columns in the NIST serum standard reference material (SRM 1957) (Table S7).

**Figure 3:**
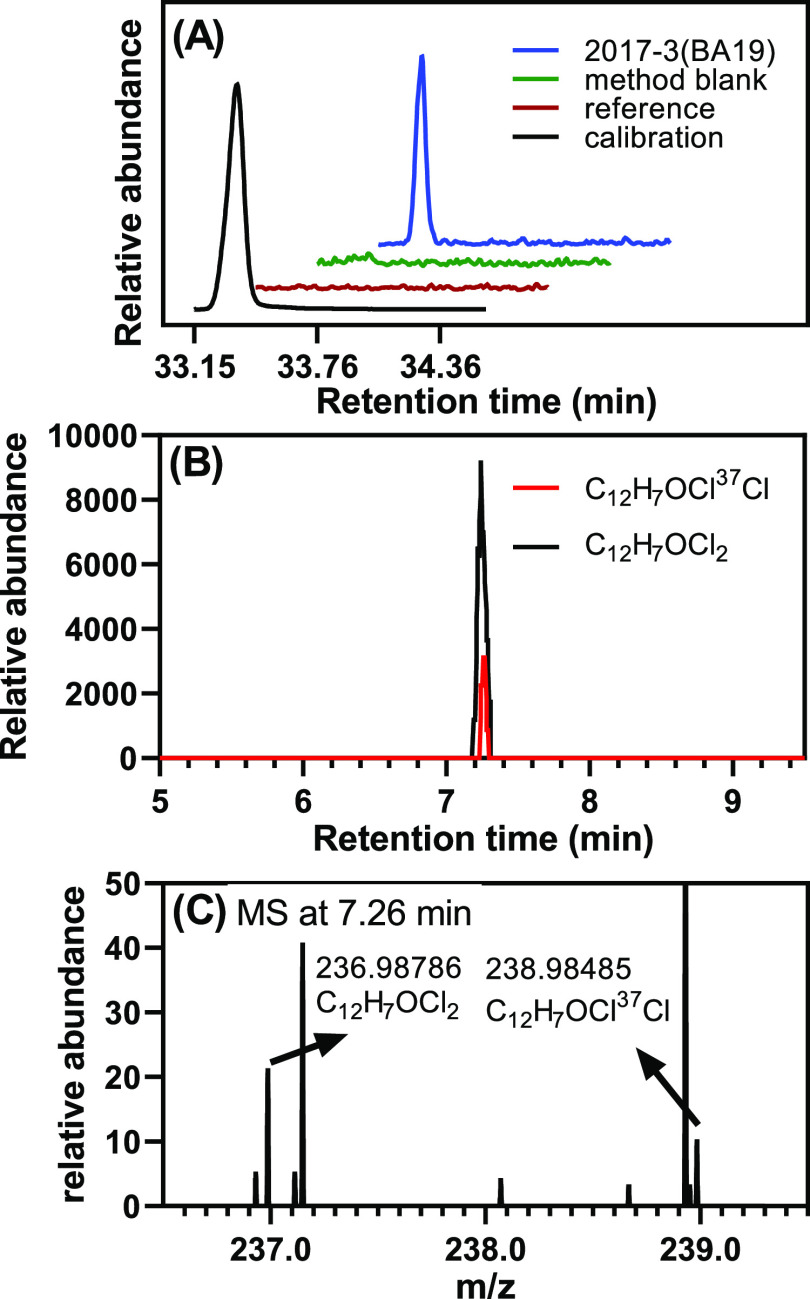
Dichlorinated OH-PCB was detected by GC–MS/MS and LC–MS Orbitrap in BA19 from a 1-day-old female donor (donor 2017-3, see Table S1). (A) GC–MS/MS chromatogram with MRM transition (*m*/*z*) of 252 → 209 showing a peak corresponding to 4′-9 and/or 4–14 (as methylated derivatives) in the extract from a BA19 sample from this donor. The calibration standard, method blank, and reference standard are shown for comparison. LC–MS Orbitrap analysis of the same brain region from the same donor also showed a single dichlorinated OH-PCB peak, indirectly confirming the presence of 4′-9 and/or4-14 in this brain sample. (B) Chromatograms extracted based on the theoretical accurate mass of the top two high-abundance isotope ions of a dichlorinated OH-PCB (chromatogram in black, [C_12_H_6_OCl_2_]^−^, *m*/*z* 236.98740, chromatogram in red, [C_12_H_6_OCl^37^Cl]^−^, *m*/*z* 238.98445) show peaks at 7.26 min. (C) Accurate mass of both ions at 7.26 min matched the theoretical accurate mass and isotopic pattern of a dichlorinated compound (1:0.6). The LC–MS Orbitrap analysis was performed in the negative polarity mode, as described in the Supporting Information.

Only a few peaks corresponding to OH-PCBs were observed in the brain sample extracts (Figures S1–S5 and Tables S11 and S12). Briefly, out of the 72 OH-PCBs analyzed, only two co-eluting congeners, 4′-9+4–14 (2,5-dichlorobiphenyl-4′-ol and/or 3,5-dichlorobiphenyl-4-ol), were detected by GC–MS/MS in 2 of the 63 samples. The highest level of these co-eluting OH-PCBs (168 pg/g ww) was detected in BA19 of a 1-day-old female donor (Figure 3). A peak corresponding to these co-eluting OH-PCBs was also observed in the hippocampus of another 1-day-old female donor using the SPB-Octyl column (Figure S1); however, their presence could not be verified on the DB-1701 column. No other OH-PCBs included in the standard mixture were detected in any brain samples. Thus, OH-PCB levels in the human brain are below the respective LODs of OH-PCBs (Table S5). Based on the MRM transition, we also observed four peaks of unknown OH-PCBs on the SPB-Octyl column and two peaks of unknown OH-PCBs on the DB-1701 column in a few samples. Peaks of one unknown monochlorinated and one unknown trichlorinated OH-PCB were observed on both columns in a few brain samples (Figures S2 and S3). Studies with authentic standards are needed to confirm that these peaks correspond to the same OH-PCB congeners.

Nt-LCMS analysis was performed for 18 brain samples in which OH-PCBs were detected in the GC–MS/MS analysis. Dichlorinated OH-PCBs were detected in five samples, and tri- and tetrachlorinated OH-PCBs were observed in all 18 samples analyzed. As described earlier, OH-PCBs were identified based on their accurate mass and isotope pattern.^37,38^ For example, a dichlorinated OH-PCB was detected in the Nt-LCMS analysis in BA19 from a 1-day-old female donor (Figure 3; donor 2017-3, see Table S1). In the GC–MS/MS analysis, we observed a peak corresponding to 4′-9+4–14 (as methylated derivatives), dichlorinated OH-PCBs, in the same brain region from this donor. Similarly, we confirmed the presence of 18 tri- and 2 tetrachlorinated OH-PCBs by GC–MS/MS and Nt-LCMS in 18 tissue samples (Figures S6–S8 and Table S18). No monochlorinated OH-PCBs were detected in the Nt-LCMS analysis. It is likely that the OH-PCBs observed in these brain regions by GC–MS/MS and in the Nt-LCMS analysis correspond to the same OH-PCB congener; however, we could not confirm this hypothesis because analytical standards are not available.

Our finding, while preliminary, is important because profiles and levels of OH-PCBs have been reported, for example, in human serum,^39,99^ breast milk,^100^ and urine.^45,46^ Typically, the major OH-PCB congeners detected in earlier studies were derived from higher chlorinated PCBs. For example, 3′-138 and 4′-130 were the predominant OH-PCBs in human postmortem liver and adipose tissues collected in 1994 in Sweden.^101^ 4–107+3–118, 3′-180, and 3′-138 were the major OH-PCBs in human postmortem adipose tissue from Spain.^102^ A few studies have reported the presence of OH-PCBs in the brain of rodents^49^ and wildlife.^103,104^ In contrast, OH-PCB profiles and levels in human brain tissue have not been investigated.

## Supporting Information Available

Sample information, OH-PCB abbreviations, LODs, levels of PCBs and OH-PCBs in NIST and brain tissue samples, and data analysis results (XLSX)
Description of chemicals and materials, extraction procedures, gas chromatographic determinations, quality assurance/quality control data, and chromatograms of OH-PCB analyses. This material is available free of charge via the internet at http://pubs.acs.org. The original GC–MS/MS data underlying this study are openly available through the Iowa Research Online repository at https://doi.org/10.25820/data.006169 (PDF)
