# Supplementary material for: Assessment of Polychlorinated Biphenyls and Their Hydroxylated Metabolites in Postmortem Human Brain Samples: Age and Brain Region Differences
Source: Environ Sci Technol. 2022 Jun 3;56(13):9515–26. doi: 10.1021/acs.est.2c00581 (PMC9260965; doi:10.1021/acs.est.2c00581)
Supplement: Supplementary file 2 — es2c00581_si_002.pdf [file es2c00581_si_002.pdf]

SUPPORTING INFORMATION

Assessment of Polychlorinated Biphenyls and Their Hydroxylated Metabolites in Postmortem Human Brain Samples: Age and Brain Region Differences

Xueshu Li,[†] *Marco M. Hefti,*[‡] *Rachel F. Marek,*[#,$] *Keri C. Hornbuckle,*[#,$]

Kai Wang,[^] *Hans-Joachim Lehmler*[*,†,$]

[†] Department of Occupational and Environmental Health, University of Iowa, Iowa City, IA 52242, USA; [‡] Department of Pathology, University of Iowa Hospital and Clinics, Iowa City, IA 52242, USA; [$] IIHR-Hydroscience and Engineering, University of Iowa, Iowa City, IA 52242, USA; [#] Department of Civil and Environmental Engineering, University of Iowa, Iowa City, IA 52242, USA; [^] Department of Biostatistics, University of Iowa, Iowa City, IA 52242, USA

Corresponding Author: Dr. Hans-Joachim Lehmler
The University of Iowa
Department of Occupational and Environmental Health
University of Iowa Research Park, #221 IREH
Iowa City, IA 52242-5000
Phone: (319) 335-4310
Fax: (319) 335-4290
e-mail: hans-joachim-lehmler@uiowa.edu

Number of Pages: 22

Number of Figures: 8

Number of Tables: 1

Table of Contents

Chemicals and materials	S3
Extraction procedure of PCBs and OH-PCBs from brain tissues for gas chromatography-tandem mass spectrometry (GC-MS/MS) analysis	S3
Targeted GC-MS/MS determination of PCBs and OH-PCBs	S5
Quality assurance/quality control for the GC-MS/MS analysis	S5
Chromatographic separation and instrument parameters for the Nt-LCMS analysis of OH-PCBs	S7
Processing of Nt-LCMS data	S7
**Figure S1.** Coeluting congeners 4'-9+4-14 (2,5-dichlorobiphenyl-4'-ol/3,5-dichlorobiphenyl-4-ol) were detected with an MRM transition of (*m/z*) 252 → 209 only in two human brain tissue samples from donors 2017-3 (BA19, see Fig. S4 in the manuscript) and 2018-18 (HC).	S8
**Figure S2.** Chromatograms on the SPB-Octyl column showing the presence of possible unknown mono- to tetra-chlorinated OH-PCB metabolites in the brain from selected donors.	S9
**Figure S3**. Chromatograms on the DB-1701 column showing the presence of unknown mono- and tri-chlorinated OH-PCB metabolites in the brain from selected donors, with MRM transitions of (*m/z*) 218 → 168 and 286 → 243, respectively.	S10
**Figure S4.** 4-107 (2,3,3',4',5-pentachlorobiphenyl-4-ol) was not detected with an MRM transition of (*m/z*) 256.9 → 340.9 in any human brain tissue samples.	S11
**Figure S5**. Coeluting congeners 5-183+4-187 (2,2',3,4,4',5',6-heptachlorobiphenyl-5-ol+2,2',3,4',5,5',6-heptachlorobiphenyl-4-ol) were not detected on GC-MS/MS with an MRM transition of (*m/z*) 423.8 → 380.8 in any human brain tissue samples.	S12
**Figure S6.** An unknown trichlorinated OH-PCB was detected by GC-MS/MS and Nt-LCMS Orbitrap in the amygdala from a 2-day old female donor (donor 2018-13, see Table S1).	S13
**Figure S7.** An unknown trichlorinated OH-PCB was detected by GC-MS/MS and Nt-LCMS Orbitrap in the BA19 from a 1-day old female donor (donor 2017-3, see Table S1).	S14
**Figure S8.** An unknown tetrachlorinated OH-PCB was detected by GC-MS/MS and Nt-LCMS Orbitrap in the amygdala from a 1-day old female donor (donor 2018-13, see Table S1).	S15
**Table SS1**. Precursor and product ions of the quantitative standards, internal standards, surrogate standards, and the corresponding collision energy.	S16
**References**	S18

Chemicals and materials. ^{13}C-labeled PCBs (Table S3) were purchased from Cambridge Isotope Laboratories Inc. (Andover, Massachusetts, USA). ^{13}C-labeled hydroxylated PCBs (OH-PCBs, Table S3) and methoxylated PCBs were obtained from Wellington Laboratories Inc. (Guelph, Ontario, Canada). Deuterated PCB 30 (d-PCB 30) was provided by C/D/N Isotopes (Pointe-Claire, Quebec, Canada). Native PCB congeners and some methoxylated PCBs were purchased from Accustandard (New Haven, Connecticut, USA). 4′-Chloro-3′-fluoro-4-hydroxy-biphenyl and the corresponding F-tagged PCB sulfate, sulfuric acid mono-(4'-chloro-3'-fluoro-biphenyl-4-yl) ammonium salt, were prepared and authenticated as described elsewhere.[1] The potassium salt of perfluorooctanesulfonic acid (Fisher Scientific, Pittsburg, Pennsylvania, USA). Pesticide grade solvents, such as hexane and dichloromethane, and mass spectrometry grade solvent, such as acetonitrile, were purchased from Fisher Scientific (Pittsburg, Pennsylvania, USA). The Standard Reference Material (SRM 1957) was provided by the National Institute of Standards and Technology (NIST, Gaithersburg, Maryland, USA).

Extraction procedure of PCBs and OH-PCBs from brain tissues for gas chromatography-tandem mass spectrometry (GC-MS/MS) analysis.[2, 3] Tissue samples were homogenized in 3 mL isopropanol, 1 mL of diethyl ether was added and samples were spiked with isotope-labeled PCB ($^{13}C_{12}$-PCB 3, $^{13}C_{12}$-PCB 15, $^{13}C_{12}$-PCB 31, $^{13}C_{12}$-PCB 52, $^{13}C_{12}$-PCB 118, $^{13}C_{12}$-PCB 153, $^{13}C_{12}$-PCB 180, ^{13}C-PCB 194, ^{13}C-PCB 206, and ^{13}C-PCB 209; 5 ng/each in hexane) and OH-PCB surrogate standards (4'-OH-[$^{13}C_{12}$]PCB 12, 4'-OH-[$^{13}C_{12}$]PCB 29, 4'-OH-[$^{13}C_{12}$]PCB 61, 4'-OH-[$^{13}C_{12}$]PCB 120, 4'-OH-[$^{13}C_{12}$]PCB 159, 4'-OH-[$^{13}C_{12}$]PCB 172, and 4-OH-[$^{13}C_{12}$]PCB 187; 5 ng/each in methanol). The tubes were inverted for 5 min, followed by centrifugation at 1,378 *g* for 5 min. The organic layer was transferred to a new tube. The residue was re-extracted with 1 mL of isopropanol and 2.5 mL of hexane and diethyl ether (9:1, vol/vol). The combined organic extracts were washed with 5 mL of phosphoric acid (0.1 M in 0.9% aqueous sodium chloride), and the aqueous phase was re-extracted with 1 mL of hexane and diethyl ether

(9:1, v/v).

The combined extract, which contained both PCBs and OH-PCBs, was concentrated to near dryness under a gentle stream of nitrogen. The extract was reconstituted in 4 mL of hexane, and 2 mL of a potassium hydroxide solution (0.5 M in water-ethanol, 1:1, v/v) was added. After inversion for 5 min and centrifugation at 1,378 *g* for 3 min, the organic phase was separated, and the bottom aqueous phase was re-extracted with 3 mL of hexane. The combined organic phase contained the PCBs. To extract the OH-PCBs, the aqueous phase was acidified with 0.5 mL of hydrochloric acid (2 M) and extracted twice with 4 mL and 3 mL of hexane and methyl t-butyl ether (9:1, vol/vol). This combined organic phase contained the OH-PCBs.

The PCB fraction was concentrated to ~ 0.5 mL under a gentle stream of nitrogen and passed through a glass cartridge filled with 2 g of acidified silica gel (silica gel and concentrated sulfuric acid, 2:1, w/w) with 0.2 g activated silica gel at the bottom and prewashed with 3 mL of hexane. PCBs were eluted from the cartridge with 14 mL of hexane. The eluent was concentrated to 4 mL and treated with 2 mL of concentrated sulfuric acid. After inversion for 5 min and centrifugation at 1,378 g for 3 min, the organic phase was collected, and the sulfuric acid layer was re-extracted with 3 mL of hexane. The combined organic extract was concentrated to about 50 µL and transferred to a glass autosampler vial with an insert. The sample was spiked with the internal standards (d_5-PCB 30 and PCB 204; 5 ng each in hexane) prior to analysis.

The OH-PCB fraction was concentrated to about 1 mL, and 5 drops of methanol were added. As described previously, the OH-PCBs were derivatized with diazomethane to the corresponding methoxylated PCBs.[4-6] After derivatization, the OH-PCB fraction was concentrated to about 0.5 mL and passed through a cartridge as described above for PCBs, except that 14 mL dichloromethane was used as eluent. The solvent was exchanged to hexane by concentrating to 0.5 mL and diluting with 3 mL of hexane three times. The extract was treated with sulfuric acid as

described above for the PCB fraction and spiked with internal standards (d_5-PCB 30 and PCB 204, 5 ng/each) prior to analysis.

Targeted GC-MS/MS determination of PCBs and OH-PCBs. PCB samples were analyzed on An Agilent GC system coupled with an Agilent 7000 Triple Quad in the multiple reaction monitoring (MRM) mode on an SPB-Octyl capillary column (30 m length, 250 µm inner diameter, 0.25 µm film thickness; Sigma-Aldrich, St. Louis, Missouri, USA). OH-PCB (as MeO-PCB) samples were analyzed on an Agilent 7890B GC system coupled with an Agilent 7000D Triple Quad in the MRM mode on SPB-Octyl column (same model as above) or DM-1701 column (30 m length, 250 µm inner diameter, 0.25 µm film thickness; Sigma-Aldrich). The following temperature program was used for all analyses on both columns: 45 °C, hold for 2 min, 100 °C/min to 75 °C, hold for 5 min, 15 °C/min to 150 °C, hold for 1 min, 2.5 °C/min to 280 °C, and hold for 5 min. The injector temperature program is as follows: start at 45 °C, hold for 0.06 min, 600 °C/min to 325 °C and hold for 5 min. The transfer line temperature was 280 °C. Helium was used as a carrier gas with a constant flow rate of 0.8 mL/min. The precursor-product ion transitions of all PCB analytes used for the MS/MS analysis have been reported previously.[7] The precursor-product ion transition of all MeO-PCB analytes for MS/MS were modified based on previous published method and are listed in Table SS1 below.[6, 8]

Quality assurance/quality control for the GC-MS/MS analysis. The brain samples were extracted and analyzed using a rigorous quality assurance/quality control (QA/QC) program established by the Analytical Core of the Iowa Superfund Research Program to assure the accuracy (analysis of SRM 1957), precision (surrogate recoveries), representativeness (analysis of sample blanks and SRM), and reproducibility (surrogate recoveries, analysis of SRM 1957) of the PCB and OH-PCB measurements (Tables S3-S7). All analyses were performed using detailed Standard Operating Procedures and use an extraction protocol that was reported previously[3] and is based on validate protocols for the analysis of PCBs and OH-PCBs in animal and human samples.[9-11]

QA/QC samples included in the analysis included primary PCB and MeO-PCB calibration standards containing 209 and 72 standards, respectively. Both standards were analyzed at the beginning and end of each set of samples to identify and quantify PCBs and OH-PCBs (as MeO-PCBs). These standards contained the PCB and MeO-PCB congeners, surrogate recovery standards, and internal quantification standards

Surrogate recovery standards (i.e., representative PCB or OH-PCB congeners) were spiked into every sample immediately prior to extraction for the primary purpose of assessing and correcting for analytical losses during sample workup and assess the precision and reproducibility of the extraction across the entire study. PCB surrogate standards included the ten ^{13}C-labeled mono- to deca-chlorinated PCBs (one from each Cl homolog) listed in Table S3. Average PCB recovery rates, including the range of recoveries and relative standard deviation for each standard, are provided in Table S3. OH-PCB surrogate standards were selected based on their commercial availability and included seven ^{13}C-labeled OH-PCBs from the di, tri, tetra, penta, hexa, and two hepta chlorinated homologs, see Table S3. Average OH-PCB recovery rates, including the range of recoveries and relative standard deviation for each standard, are provided in Table S3.

Based on our earlier studies, deuterated PCB 30 and PCB 204 were selected as representative congeners to normalize instrument response for PCB and MeO-PCB congeners to concentrations in the calibration standard. These internal quantification standards were spiked into every sample immediately prior to instrument analysis.

Standard Reference Materials SRM 1957 (organic contaminants in non-fortified human serum, National Institute of Standards and Technology) was used to assess the accuracy. The SRM was selected because no suitable SRM or laboratory reference material is currently available for human brain tissues. A comparison of the measured and certified PCB and published OH-PCB levels in this SRM is provided in Table S6. Moreover, a SRM 1957 sample was extracted with every sample

batch to evaluate the reproducibility of the analysis. The results from all SRM samples extracted as part of this study are also summarized in Table S6.

The congener-specific analysis of PCB and OH-PCBs (as MeO-PCBs) was performed on a triple quadrupole GC-MS-MS using the internal standard method to identify and quantify all 209 PCBs and 72 OH-PCBs as MeO-PCBs in a variety of environmental matrices. Past examples have included air,[6, 12-17] sediment and soil,[18-22] water and porewater,[23, 24] and human serum.[2, 25-28]

Chromatographic separation and instrument parameters used for the Nt-LCMS analysis of OH-PCBs. Water and acetonitrile were used as mobile phases A and B, respectively, with a mobile flow rate of 0.3 mL/min and a pressure of 4000-8000 psi at 25 °C. Both mobile phases contained 10 mM ammonium formate and 0.1% (v/v) formic acid. The UPLC gradient program was as follows: starting at 5% B, held for 1 min, increased linearly to 95% B, held for 3 min, and returning to 5% B, with a hold for 4 min before the next injection. The injection volume was 5 μL. The Q-Exactive Orbitrap Mass Spectrometer (Thermo Fisher Scientific, Waltham, MA, USA) was operated in the negative polarity mode. The source parameters settings were as follows: spray voltage, 2472 V; spray current, 18.2 μA; capillary temperature, 256 °C; sheath gas flow rate, 48; auxiliary gas flow rate, 2; and auxiliary temperature, 413 °C. The analyses were performed in the full scan mode with a range from 85 to 1275 *m/z*. The Automatic Gain Control target setting was 1×10^6, the full scan resolution setting was 70,000, and the maximum interval time (IT) was 200 ms.

Processing of Nt-LCMS data. The chromatographic peaks of suspected OH-PCBs were extracted from the acquired data (as .raw file) with Thermo Xcalibur (version 4.1, Thermo Fisher Scientific, USA). The chromatogram extraction was performed with a *m/z* tolerance of 5 ppm, mass precision decimals of 5, and the smoothing factor of 7. The isotopic pattern of chlorine was used as an important factor for confirming putative OH-PCBs, as described.[29-31] The

chromatographic data of the detected OH-PCBs were exported and figures were prepared with GraphPad Prism (version 9.0, GraphPad Software, USA).

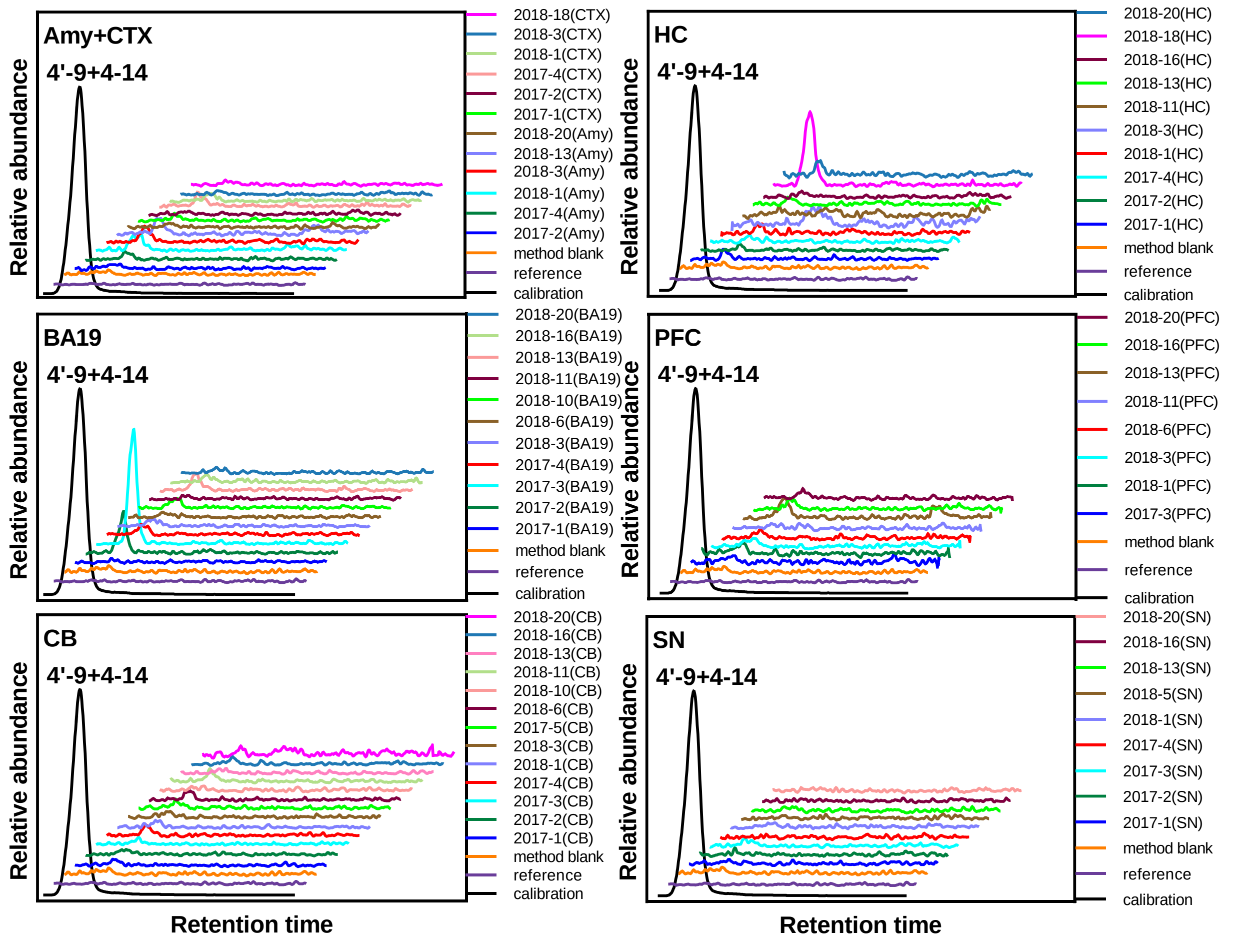

Figure S1. Coeluting congeners 4'-9+4-14 (2,5-dichlorobiphenyl-4'-ol and 3,5-dichlorobiphenyl-4-ol) were detected with an MRM transition of (*m/z*) 252 → 209 only in two human brain tissue samples from donors 2017-3 (BA19) and 2018-18 (HC). Chromatograms of the corresponding calibration and reference standards to assess impurities in the analytical standards and a blank sample to control for background contamination are shown for comparison. Analyses were performed by GC-MS/MS on the SPB-Octyl column as described in the Experimental section. For more information regarding the donors, see Table S1. Amy: amygdala, BA19: Broadman area 19, CB: cerebellum, CTX: cortex, HC: Hippocampus, PFC: prefrontal cortex SN: subtantia nigra.

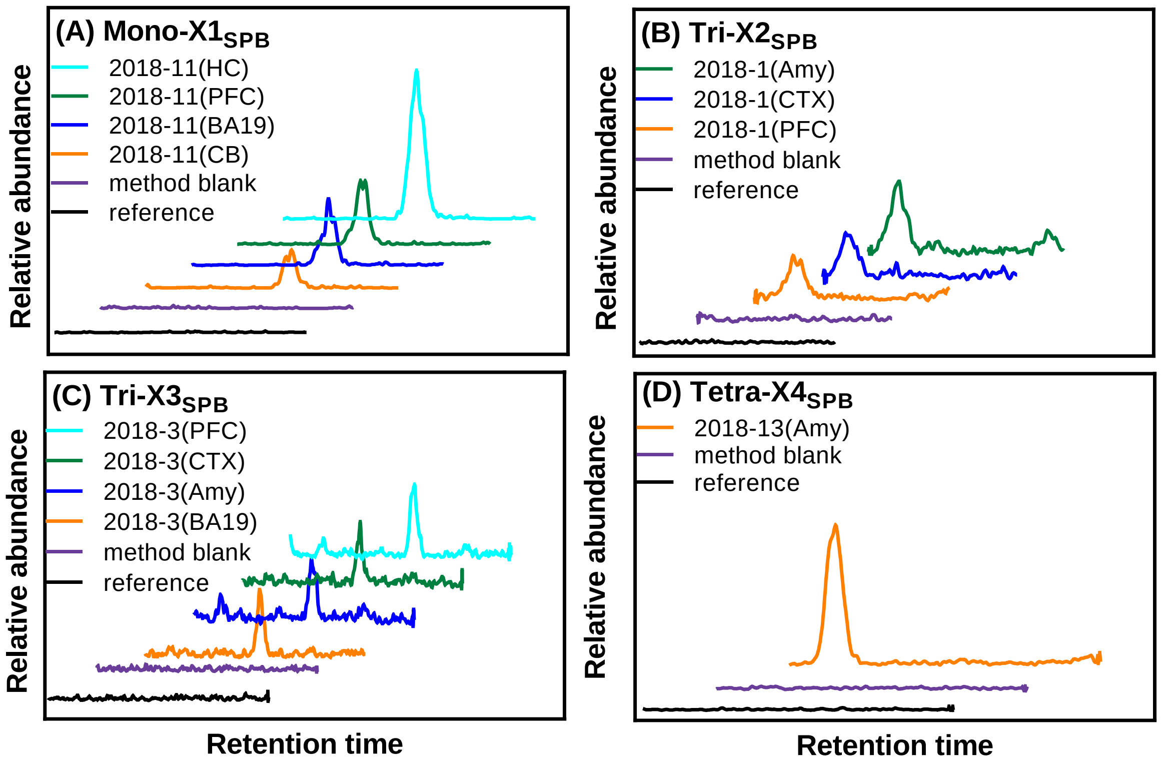

Figure S2. Chromatograms on the SPB-Octyl column showing the presence of possible unknown mono- to tetra-chlorinated OH-PCB metabolites in the brain from selected donors: (A) Mono-X1 in brain regions from donor 2018-11 (1-year-old female) with an MRM transition (*m/z*) 218 → 168; (B) Tri-X2 in brain regions from donor 2018-1 (60-year-old female) with an MRM transition (*m/z*) 286 → 243; (C) Tri-X3 in brain regions from donor 2018-3 (1-day-old female) with an MRM transition (*m/z*) 286 → 243; and (D) Tetra-X4 in the amygdala from donor 2018-13 (2-day-old female) with an MRM transition 321.9 → 278.9. Chromatograms of the corresponding calibration and reference standards to assess impurities in the analytical standards and a blank sample to control for background contamination are shown for comparison. Analyses were performed by GC-MS/MS on the SPB-Octyl column as described in the Experimental section. For more information regarding the donors, see Table S1. Amy: amygdala, BA19: Broadman area 19, CTX: cortex, PFC: prefrontal cortex.

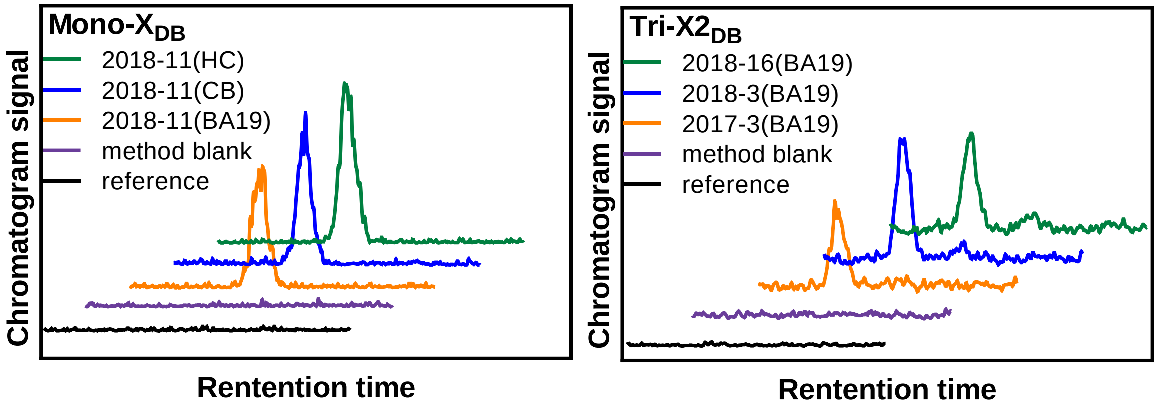

Figure S3. Chromatograms on the DB-1701 column showing the presence of unknown mono- and tri-chlorinated OH-PCB metabolites in the brain from selected donors, with MRM transitions of (m/z) 218 → 168 and 286 → 243, respectively. Chromatograms of the corresponding calibration and reference standards to assess impurities in the analytical standards and a blank sample to control for background contamination are shown for comparison. Analyses were performed by GC-MS/MS on the DB-1701 column as described in the Experimental section. For more information regarding the donors, see Table S1. BA19: Broadman area 19, CB: cerebellum, HC: hippocampus.

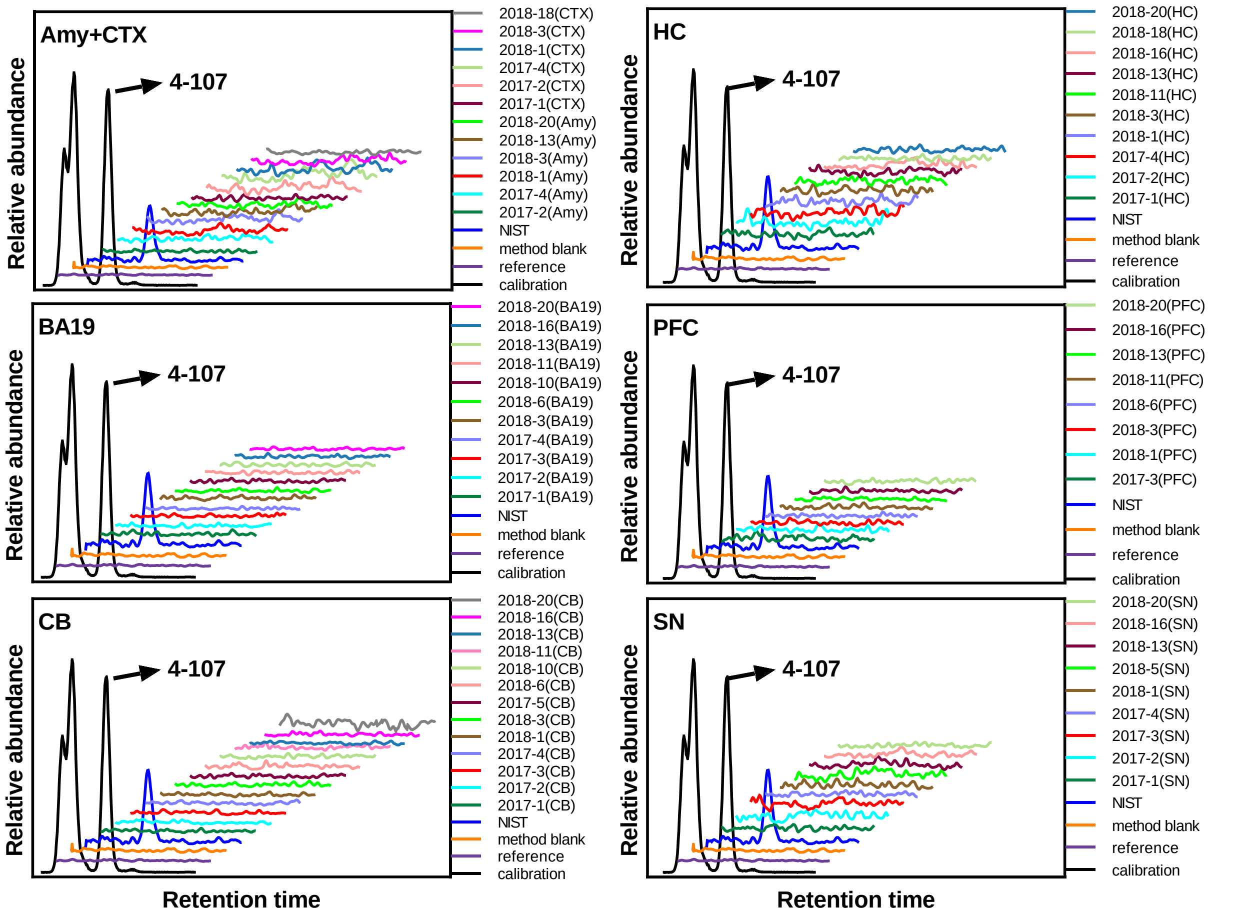

Figure S4. 4-107 (2,3,3',4',5-pentachlorobiphenyl-4-ol) was not detected with an MRM transition of (*m/z*) 256.9 → 340.9 in any human brain tissue samples. However, 4-107 was detected in the NIST standard analyzed in parallel and has been detected with a high detection frequency in serum samples from the AESOP (Airborne Exposures to Semi-volatile Organic Pollutants) study.[2] Chromatograms of the corresponding calibration and reference standards to assess impurities in the analytical standards and a blank sample to control for background contamination are shown for comparison. Analyses were performed by GC-MS/MS on the SPB-Octyl column as described in the Experimental section. For more information regarding the donors, see Table S1. Amy: amygdala, BA19: Broadman area 19, CB: cerebellum, CTX: cortex, HC: Hippocampus, PFC: prefrontal cortex SN: subtantia nigra.

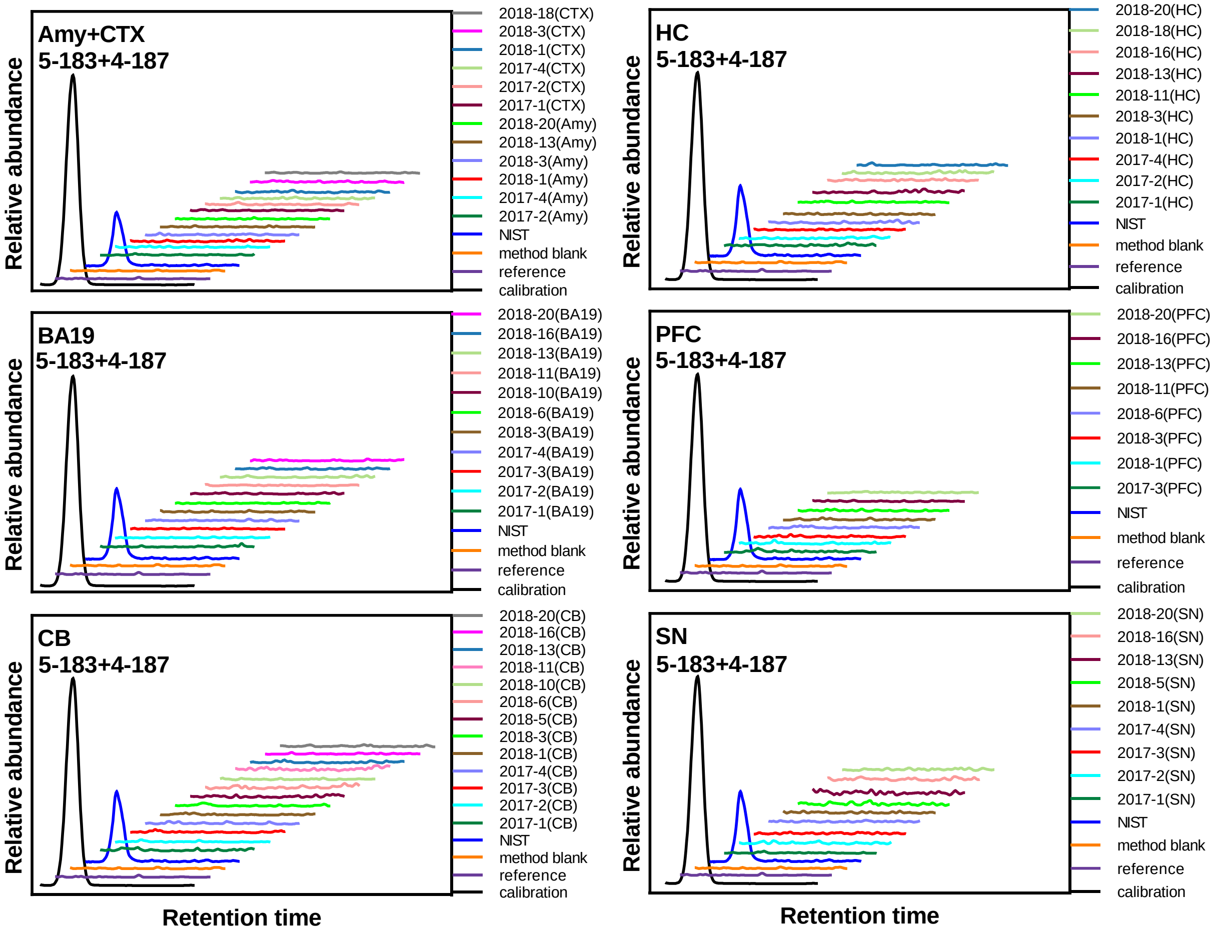

Figure S5. Coeluting congeners 5-183+4-187 (2,2',3,4,4',5',6-heptachlorobiphenyl-5-ol + 2,2',3,4',5,5',6-heptachlorobiphenyl-4-ol) were not detected on GC-MS/MS with an MRM transition of (*m/z*) 423.8 → 380.8 in any human brain tissue samples. However, 5-183+4-187 was detected in the NIST standard analyzed in parallel and 4-187 has been detected with a high detection frequency in serum samples from the AESOP (Airborne Exposures to Semi-volatile Organic Pollutants) study.[2] Chromatograms of the corresponding calibration and reference standards to assess impurities in the analytical standards and a blank sample to control for background contamination are shown for comparison. Analyses were performed by GC-MS/MS on the SPB-Octyl column as described in the Experimental section. For more information regarding the donors, see Table S1. Amy: amygdala, BA19: Broadman area 19, CB: cerebellum, CTX: cortex, HC: Hippocampus, PFC: prefrontal cortex SN: subtantia nigra.

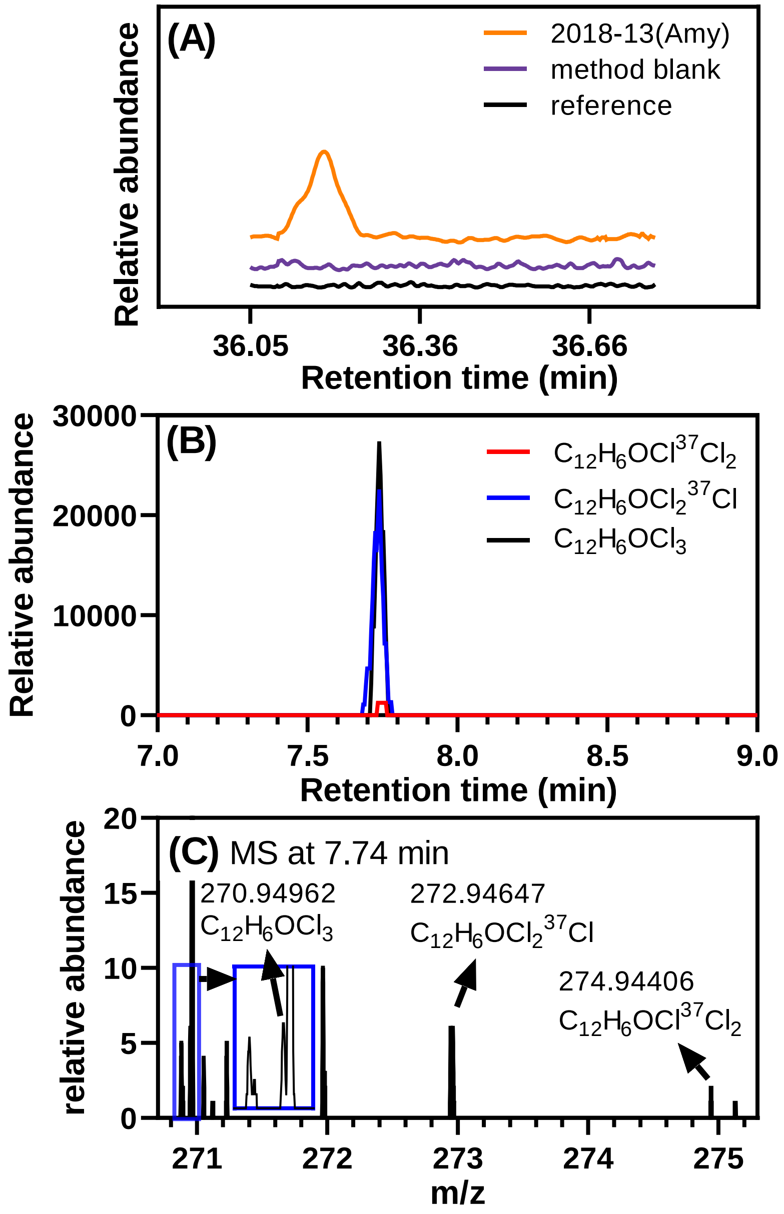

Figure S6. An unknown trichlorinated OH-PCB was detected by GC-MS/MS and Nt-LCMS Orbitrap in the amygdala from a 2-day old female donor (donor 2018-13, see Table S1). (A) GC-MS/MS chromatogram with MRM transition (*m/z*) of 286 → 243 showing a peak corresponding to a trichlorinated OH-PCB (as methoxylated PCBs) in the extract from an amygdala sample from this donor. The method blank, and reference standard are shown for comparison. Nt-LCMS Orbitrap analysis of the same brain region from the same donor also showed a single trichlorinated OH-PCB peak, indirectly confirming the presence of a trichlorinated OH-PCB in this brain sample. (B) Chromatograms extracted based on the theoretical accurate mass of the top three high abundance isotope ions of a trichlorinated OH-PCB (chromatogram in black, $[C_{12}H_6OCl_3]^-$, *m/z* 270.94843, chromatogram in blue, $[C_{12}H_6OCl_2{}^{37}Cl]^-$, *m/z* 272.94548, chromatogram in red, $[C_{12}H_6OCl^{37}Cl_2]^-$, *m/z* 274.94253) show peaks at 7.74 min. (C) The accurate mass of both ions at 7.74 min matched the theoretical accurate mass and isotopic pattern of a trichlorinated compound (1 : 1 : 0.3). The Nt-LCMS Orbitrap analysis was performed in the negative polarity mode as described in the Supporting Information.

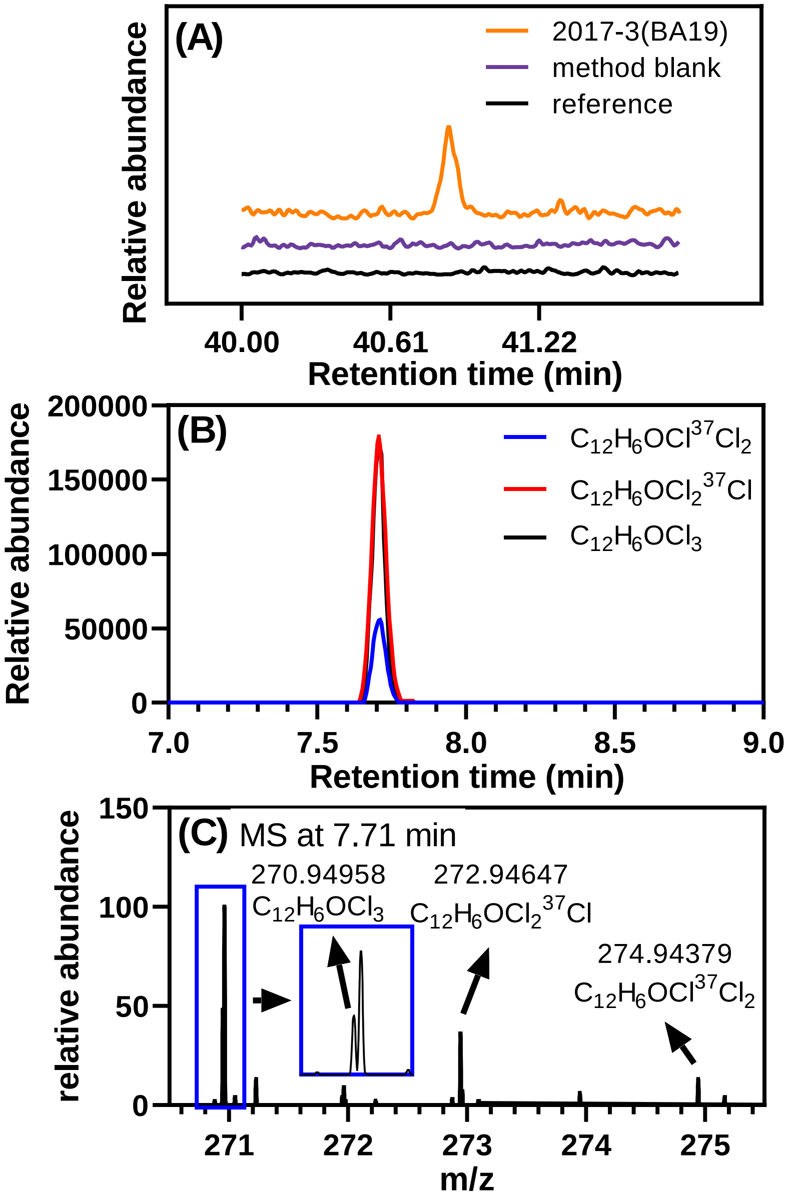

Figure S7. An unknown trichlorinated OH-PCB was detected by GC-MS/MS and Nt-LCMS Orbitrap in the BA19 from a 1-day old female donor (donor 2017-3, see Table S1). (A) GC-MS/MS chromatogram with MRM transition (*m*/*z*) of 286 → 243 showing a peak corresponding to a trichlorinated OH-PCB (as methoxylated PCBs) in the extract from an amygdala sample from this donor. The method blank and reference standard are shown for comparison. Nt-LCMS Orbitrap analysis of the same brain region from the same donor also showed a single trichlorinated OH-PCB peak, indirectly confirming the presence of a trichlorinated OH-PCB in this brain sample. (B) Chromatograms extracted based on the theoretical accurate mass of the top three high abundance isotope ions of a trichlorinated OH-PCB (chromatogram in black, $[C_{12}H_6OCl_3]^-$, *m/z* 270.94843, chromatogram in blue, $[C_{12}H_6OCl_2{}^{37}Cl]^-$, *m/z* 272.94548, chromatogram in red, $[C_{12}H_6OCl^{37}Cl_2]^-$, *m/z* 274.94253) show peaks at 7.74 min. (C) The accurate mass of both ions at 7.74 min matched the theoretical accurate mass and isotopic pattern of a trichlorinated compound (1 : 1 : 0.3). The Nt-LCMS Orbitrap analysis was performed in the negative polarity mode as described in the Supporting Information.

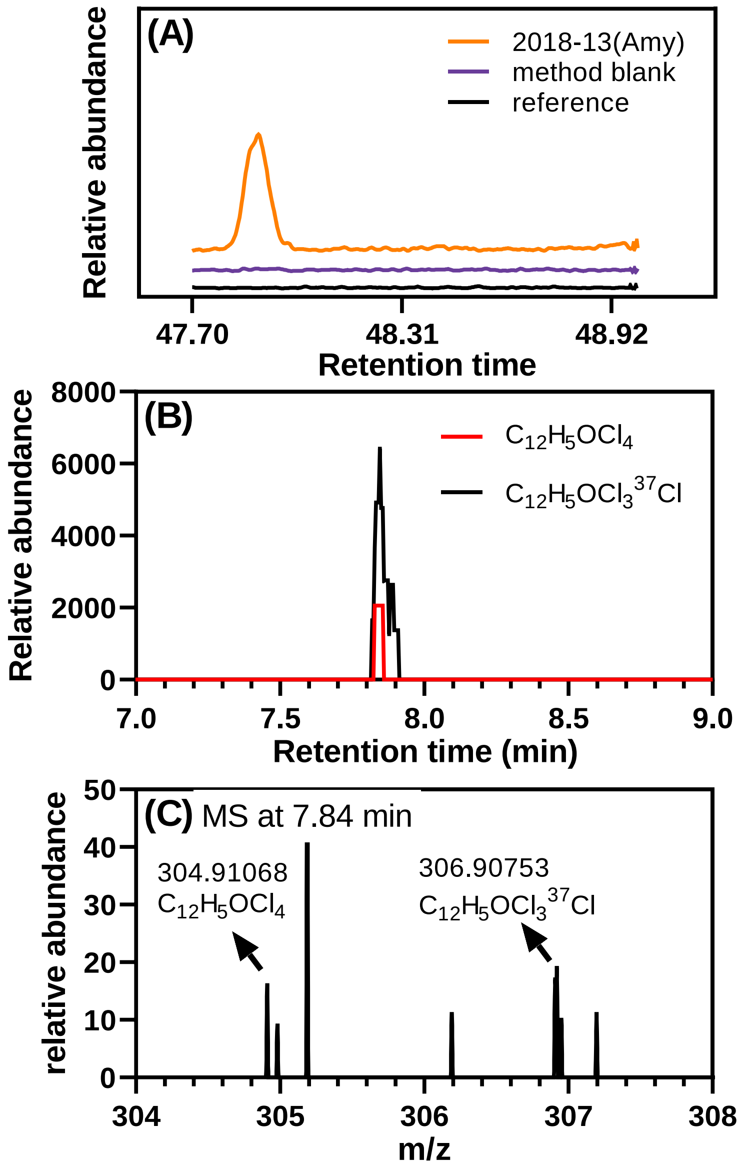

Figure S8. An unknown tetrachlorinated OH-PCB was detected by GC-MS/MS and Nt-LCMS Orbitrap in the amygdala from a 1-day old female donor (donor 2018-13, see Table S1). (A) GC-MS/MS chromatogram with MRM transition (*m/z*) of 321.9 → 278.9 showing a peak corresponding to a tetrachlorinated OH-PCB (as methoxylated PCBs) in the extract from an amygdala sample from this donor. The method blank, and reference standard are shown for comparison. Nt-LCMS Orbitrap analysis of the same brain region from the same donor also showed a single tetrachlorinated OH-PCB peak, indirectly confirming the presence of a tetrachlorinated OH-PCB in this brain sample. (B) Chromatograms extracted based on the theoretical accurate mass of the top two high abundance isotope ions of a dichlorinated OH-PCB (chromatogram in black, $[C_{12}H_5OCl_4]^-$, *m/z* 304.90946, chromatogram in red, $[C_{12}H_5OCl_3{}^{37}Cl]^-$, *m/z* 306.90651) show peaks at 7.84 min. (C) The accurate mass of both ions at 7.84 min matched the theoretical accurate mass and isotopic pattern of a tetrachlorinated compound (0.8 : 1). The Nt-LCMS Orbitrap analysis was performed in the negative polarity mode as described in the Supporting Information.

Table SS1. Precursor and product ions of the quantitative standards, internal standards, surrogate standards, and the corresponding collision energy.[6, 32]

Abbreviation	Homolog	Precursor Ion (*m/z*)	Product Ion (*m/z*)	Collision Energy (eV)
4-MeO-PCB 1	mono	218.0	175.0	20
2'-MeO-PCB 2	mono	218.0	168.1	30
3'-MeO-PCB 2	mono	218.0	188.0	15
4'-MeO-PCB 2	mono	218.0	175.0	25
2-MeO-PCB 2	mono	218.0	168.1	30
6-MeO-PCB 2	mono	218.0	168.1	30
5-MeO-PCB 2	mono	218.0	152.1	40
4-MeO-PCB 2	mono	218.0	203.0	15
4'-MeO-PCB 3	mono	218.0	175.0	25
2'-MeO-PCB 5	di	252.0	202.0	35
3'-MeO-PCB 9	di	252.0	152.0	35
4'-MeO-PCB 9	di	252.0	209.0	20
2'-MeO-PCB 12	di	252.0	202.0	30
4-MeO-PCB 14	di	252.0	237.0	15
4'-MeO-PCB 18	tri	286.0	243.0	25
6'-MeO-PCB 26	tri	286.0	236.0	30
4'-MeO-PCB 26	tri	286.0	243.0	25
2'-MeO-PCB 30	tri	286.0	236.0	30
3'-MeO-PCB 30	tri	286.0	186.0	35
4'-MeO-PCB 30	tri	286.0	243.0	25
3-MeO-PCB 54	tetra	321.9	278.9	20
2'-MeO-PCB 61	tetra	321.9	271.9	25
3'-MeO-PCB 61	tetra	321.9	291.9	20
4'-MeO-PCB 61	tetra	321.9	278.9	25
2'-MeO-PCB 65	tetra	321.9	271.9	25
3'-MeO-PCB 65	tetra	321.9	222.0	40
4'-MeO-PCB 65	tetra	321.9	278.9	25
4-MeO-PCB 65	tetra	321.9	278.9	25
6'-MeO-PCB 69	tetra	321.9	271.9	25
4'-MeO-PCB 69	tetra	321.9	278.9	25
4'-MeO-PCB 72	tetra	321.9	306.9	15
4'-MeO-PCB 79	tetra	321.9	306.9	20
6'-MeO-PCB 83	penta	355.9	305.9	25
4'-MeO-PCB 86	penta	355.9	312.9	25
4'-MeO-PCB 93	penta	355.9	255.9	40
4'-MeO-PCB 97	penta	355.9	312.9	25
6'-MeO-PCB 101	penta	355.9	305.9	20
4'-MeO-PCB 101	penta	355.9	312.9	25
2'-MeO-PCB 106	penta	355.9	305.9	30
4-MeO-PCB 107	penta	355.9	340.9	15
4'-MeO-PCB 108	penta	355.9	340.9	15
2'-MeO-PCB 114	penta	355.9	305.9	30
3-MeO-PCB 118	penta	355.9	312.9	25
4'-MeO-PCB 120	penta	355.9	340.9	15

Table SS1—continued. Precursor and product ions of the quantitative standards, internal standards, surrogate standards, and the corresponding collision energy.

4'-MeO-PCB127	penta	355.9	340.9	20
4'-MeO-PCB 130	hexa	389.9	346.8	25
4-MeO-PCB 134	hexa	389.9	346.8	25
3'-MeO-PCB 138	hexa	389.9	346.8	25
5-MeO-PCB 138	hexa	389.9	346.8	25
4-MeO-PCB 146	hexa	389.9	346.8	25
3,3'-di-MeO-PCB 155	Hexa	419.9	404.8	15
4'-MeO-PCB 159	hexa	389.9	374.8	15
4-MeO-PCB 162	hexa	389.9	374.8	15
4-MeO-PCB 163	hexa	389.9	346.8	25
4'-MeO-PCB 172	hepta	423.8	380.8	25
4'-MeO-PCB 172	hepta	423.8	380.8	25
4-MeO-PCB 177	hepta	423.8	380.8	25
4-MeO-PCB 178	hepta	423.8	380.8	25
3'-MeO-PCB 180	hepta	423.8	380.8	25
3'-MeO-PCB 182	hepta	423.8	408.8	10
3'-MeO-PCB 183	hepta	423.8	380.8	25
5-MeO-PCB 183	hepta	423.8	380.8	25
3'-MeO-PCB 184	hepta	423.8	408.8	15
4-MeO-PCB 187	hepta	423.8	380.8	25
4'-MeO-PCB 198	octa	459.8	416.8	25
4'-MeO-PCB 199	octa	459.8	416.8	25
4'-MeO-PCB 200	octa	459.8	416.8	25
4'-MeO-PCB 201	octa	459.8	416.8	25
4-MeO-PCB 202	octa	459.8	416.8	25
4,4'-di-MeO-PCB 202	octa	489.8	446.8	20
3'-MeO-PCB 203	octa	459.8	416.8	25
4'-MeO-PCB 208	nona	493.7	450.7	30
**Internal standards and surrogate standards**				
d_5-PCB 30	tri	261.0	190.9	30
PCB 204	octa	429.8	357.8	35
$^{13}C_{12}$-4'-MeO-PCB 12	di	264.0	220.0	30
$^{13}C_{12}$-4'-MeO-PCB 29	tri	298.0	254.0	25
$^{13}C_{12}$-4'-MeO-PCB 61	tetra	334.0	289.9	25
$^{13}C_{12}$-4'-MeO-PCB 120	penta	367.9	352.9	15
$^{13}C_{12}$-4'-MeO-PCB 159	hexa	401.9	386.9	15
$^{13}C_{12}$-4'-MeO-PCB 172	hepta	435.9	391.8	30
$^{13}C_{12}$-4-MeO-PCB 187	hepta	435.9	391.8	25

The short names of the MeO-PCB congeners are based on the PCB congener table of the US EPA. The number indicating the position of the methoxy group is based on the numbering scheme of the respective PCB congener, as proposed previously.[33]

References:

1. Dhakal, K.; He, X.; Lehmler, H.-J.; Teesch, L. M.; Duffel, M. W.; Robertson, L. W., Identification of sulfated metabolites of 4-chlorobiphenyl (PCB3) in the serum and urine of male rats. *Chem. Res. Toxicol.* **2012,** *25*, (12), 2796-2804.
2. Marek, R. F.; Thorne, P. S.; DeWall, J.; Hornbuckle, K. C., Variability in PCB and OH-PCB serum levels in children and their mothers in urban and rural U.S. communities. *Environ. Sci. Technol.* **2014,** *48*, (22), 13459-13467.
3. Milanowski, B.; Lulek, J.; Lehmler, H.-J.; Kania-Korwel, I., Assessment of the disposition of chiral polychlorinated biphenyls in female mdr 1a/b knockout versus wild-type mice using multivariate analyses. *Environ. Int.* **2010,** *36*, (8), 884-892.
4. Li, X.; Wu, X.; Kelly, K. M.; Veng-Pedersen, P.; Lehmler, H. J., Toxicokinetics of chiral PCB 136 and its hydroxylated metabolites in mice with a liver-specific deletion of cytochrome p450 reductase. *Chem. Res. Toxicol.* **2019,** *32*, (4), 727-736.
5. Kania-Korwel, I.; Vyas, S. M.; Song, Y.; Lehmler, H. J., Gas chromatographic separation of methoxylated polychlorinated biphenyl atropisomers. *J. Chromatogr. A* **2008,** *1207*, (1-2), 146-154.
6. Marek, R. F.; Thorne, P. S.; Herkert, N. J.; Awad, A. M.; Hornbuckle, K. C., Airborne PCBs and OH-PCBs inside and outside urban and rural U.S. schools. *Environ. Sci. Technol.* **2017,** *51*, (14), 7853-7860.
7. Bannavti, M. K.; Jahnke, J. C.; Marek, R. F.; Just, C. L.; Hornbuckle, K. C., Room-to-room variability of airborne polychlorinated biphenyls in schools and the application of air sampling for targeted source evaluation. *Environ. Sci. Technol.* **2021,** *55*, (14), 9460-9468.

8. Marek, R. F.; Martinez, A.; Hornbuckle, K. C., Discovery of hydroxylated polychlorinated biphenyls (OH-PCBs) in sediment from a Lake Michigan waterway and original commercial Aroclors. *Environ. Sci. Technol.* **2013,** *47*, (15), 8204-8210.
9. Kania-Korwel, I.; Zhao, H.; Norstrom, K.; Li, X.; Hornbuckle, K. C.; Lehmler, H.-J., Simultaneous extraction and clean-up of PCBs and their metabolites from small tissue samples using pressurized liquid extraction. *J. Chromatogr. A* **2008,** *1214*, 37-46.
10. Hovander, L.; Linderholm, L.; Athanasiadou, M.; Athanassiadis, I.; Bignert, A.; Faengstroem, B.; Kocan, A.; Petrik, J.; Trnovec, T.; Bergman, A., Levels of PCBs and their metabolites in the serum of residents of a highly contaminated area in eastern Slovakia. *Environ. Sci. Technol.* **2006,** *40*, (12), 3696-3703.
11. Hovander, L.; Athanasiadou, M.; Asplund, L.; Jensen, S.; Wehler, E. K., Extraction and cleanup methods for analysis of phenolic and neutral organohalogens in plasma. *J. Anal. Toxicol.* **2000,** *24*, (8), 696-703.
12. Herkert, N. J.; Spak, S. N.; Smith, A.; Schuster, J. K.; Harner, T.; Martinez, A.; Hornbuckle, K. C., Calibration and evaluation of PUF-PAS sampling rates across the Global Atmospheric Passive Sampling (GAPS) network. *Environ. Sci. Process. Impacts* **2018,** *20*, (1), 210-219.
13. Herkert, N. J.; Jahnke, J. C.; Hornbuckle, K. C., Emissions of tetrachlorobiphenyls (PCBs 47, 51, and 68) from polymer resin on kitchen cabinets as a non-Aroclor source to residential air. *Environ. Sci. Technol.* **2018,** *52*, (9), 5154-5160.
14. Martinez, A.; Hadnott, B. N.; Awad, A. M.; Herkert, N. J.; Tomsho, K.; Basra, K.; Scammell, M. K.; Heiger-Bernays, W.; Hornbuckle, K. C., Release of airborne polychlorinated biphenyls from new bedford harbor results in elevated concentrations in the surrounding air. *Environ. Sci. Technol. Lett.* **2017,** *4*, (4), 127-131.

15. Awad, A. M.; Martinez, A.; Marek, R. F.; Hornbuckle, K. C., Occurrence and distribution of two hydroxylated polychlorinated biphenyl congeners in Chicago air. *Environ. Sci. Technol. Lett.* **2016,** *3*, (2), 47-51.

16. Ampleman, M. D.; Martinez, A.; DeWall, J.; Rawn, D. F. K.; Hornbuckle, K. C.; Thorne, P. S., Inhalation and dietary exposure to PCBs in urban and rural cohorts via congener-specific measurements. *Environ. Sci. Technol.* **2015,** *49*, (2), 1156-1164.

17. Hu, D. F.; Lehmler, H. J.; Martinez, A.; Wang, K.; Hornbuckle, K. C., Atmospheric PCB congeners across Chicago. *Atmos. Environ.* **2010,** *44*, (12), 1550-1557.

18. Martinez, A.; Erdman, N. R.; Rodenburg, Z. L.; Eastling, P. M.; Hornbuckle, K. C., Spatial distribution of chlordanes and PCB congeners in soil in Cedar Rapids, Iowa, USA. *Environ. Pollut.* **2012,** *161*, 222-228.

19. Martinez, A.; Hornbuckle, K. C., Record of PCB congeners, sorbents and potential toxicity in core samples in Indiana Harbor and Ship Canal. *Chemosphere* **2011,** *85*, (3), 542-547.

20. Martinez, A.; Norstrom, K.; Wang, K.; Hornbuckle, K. C., Polychlorinated biphenyls in the surficial sediment of Indiana Harbor and Ship Canal, Lake Michigan. *Environ. Int.* **2010,** *36*, (8), 849-854.

21. Martinez, A.; Schnoebelen, D. J.; Hornbuckle, K. C., Polychlorinated biphenyl congeners in sediment cores from the Upper Mississippi River. *Chemosphere* **2016,** *144*, 1943-1949.

22. Peverly, A. A.; O'Sullivan, C.; Liu, L. Y.; Venier, M.; Martinez, A.; Hornbuckle, K. C.; Hites, R. A., Chicago's Sanitary and Ship Canal sediment: Polycyclic aromatic hydrocarbons, polychlorinated biphenyls, brominated flame retardants, and organophosphate esters. *Chemosphere* **2015,** *134*, 380-386.

23. Martinez, A.; O'Sullivan, C.; Reible, D.; Hornbuckle, K. C., Sediment pore water distribution coefficients of PCB congeners in enriched black carbon sediment. *Environ. Pollut.* **2013,** *182*, 357-363.

24. Martinez, A.; Wang, K.; Hornbuckle, K. C., Fate of PCB congeners in an industrial harbor of Lake Michigan. *Environ. Sci. Technol.* **2010,** *44,* (8), 2803-2808.

25. Koh, W. X.; Hornbuckle, K. C.; Marek, R. F.; Wang, K.; Thorne, P. S., Hydroxylated polychlorinated biphenyls in human sera from adolescents and their mothers living in two US Midwestern communities. *Chemosphere* **2016,** *147,* 389-395.

26. Koh, W. X.; Hornbuckle, K. C.; Thorne, P. S., Human serum from urban and rural adolescents and their mothers shows exposure to polychlorinated biphenyls not found in commercial mixtures. *Environ. Sci. Technol.* **2015,** *49,* (13), 8105-8112.

27. Koh, W. X.; Hornbuckle, K. C.; Wang, K.; Thorne, P. S., Serum polychlorinated biphenyls and their hydroxylated metabolites are associated with demographic and behavioral factors in children and mothers. *Environ. Int.* **2016,** *94*, 538-545.

28. Marek, R. F.; Thorne, P. S.; Wang, K.; Dewall, J.; Hornbuckle, K. C., PCBs and OH-PCBs in serum from children and mothers in urban and rural U.S. communities. *Environ. Sci. Technol.* **2013,** *47,* (7), 3353-3361.

29. Zhang, C. Y.; Flor, S.; Ruiz, P.; Ludewig, G.; Lehmler, H. J., Characterization of the metabolic pathways of 4-chlorobiphenyl (PCB3) in HepG2 cells using the metabolite profiles of its hydroxylated metabolites. *Environ. Sci. Technol.* **2021,** *55,* (13), 9052-9062.

30. Li, X.; Liu, Y.; Martin, J. W.; Cui, J. Y.; Lehmler, H.-J., Nontarget analysis reveals gut microbiome-dependent differences in the fecal PCB metabolite profiles of germ-free and conventional mice. *Environ. Poll.* **2021**, 115726.

31. Zhang, C.-Y.; Flor, S.; Ruiz, P.; Dhakal, R.; Hu, X.; Teesch, L. M.; Ludewig, G.; Lehmler, H.-J., 3,3′-Dichlorobiphenyl is metabolized to a complex mixture of oxidative metabolites, including novel methoxylated metabolites, by HepG2 cells. *Environ. Sci. Technol.* **2020,** *55*, (13), 9052-9062.

32. Saktrakulkla, P.; Dhakal, R. C.; Lehmler, H. J.; Hornbuckle, K. C., A semi-target analytical method for quantification of OH-PCBs in environmental samples. *Environ. Sci. Poll. Res.* **2020,** *27*, (9), 8859-8871.

33. Maervoet, J.; Covaci, A.; Schepens, P.; Sandau, C. D.; Letcher, R. J., A reassessment of the nomenclature of polychlorinated biphenyl (PCB) metabolites. *Environ. Health Perspect.* **2004,** *112*, (3), 291-4.
